# Systematic Review Looking at the Use of Technology to Measure Free-Living Symptom and Activity Outcomes in Parkinson’s Disease in the Home or a Home-like Environment

**DOI:** 10.3233/JPD-191781

**Published:** 2020-04-03

**Authors:** Catherine Morgan, Michal Rolinski, Roisin McNaney, Bennet Jones, Lynn Rochester, Walter Maetzler, Ian Craddock, Alan L. Whone

**Affiliations:** a Translational Health Sciences, Bristol Medical School, University of Bristol, Bristol, UK; b School of Computer Science, Electrical and Electronic Engineering, and Engineering Mathematics, Faculty of Engineering, University of Bristol, Bristol, UK; c Movement Disorders Group, Bristol Brain Centre, Southmead Hospital, North Bristol National Health Service Trust, Bristol, UK; d Library and Knowledge Service, Learning and Research, Southmead Hospital, North Bristol National Health Service Trust, Bristol, UK; e Institute of Neuroscience, Newcastle University, Newcastle Upon Tyne, UK; f Newcastle Upon Tyne Hospitals National Health Service Foundation Trust, Newcastle Upon Tyne, UK; g Department of Neurology, Christian-Albrechts University, Kiel, Germany

**Keywords:** Parkinsonian disorders, basal ganglia diseases, technology, algorithms, patient outcome assessment

## Abstract

**Background:**

The emergence of new technologies measuring outcomes in Parkinson’s disease (PD) to complement the existing clinical rating scales has introduced the possibility of measurement occurring in patients’ own homes whilst they freely live and carry out normal day-to-day activities.

**Objective:**

This systematic review seeks to provide an overview of what technology is being used to test which outcomes in PD from free-living participant activity in the setting of the home environment. Additionally, this review seeks to form an impression of the nature of validation and clinimetric testing carried out on the technological device(s) being used.

**Methods:**

Five databases (Medline, Embase, PsycInfo, Cochrane and Web of Science) were systematically searched for papers dating from 2000. Study eligibility criteria included: adults with a PD diagnosis; the use of technology; the setting of a home or home-like environment; outcomes measuring any motor and non-motor aspect relevant to PD, as well as activities of daily living; unrestricted/unscripted activities undertaken by participants.

**Results:**

65 studies were selected for data extraction. There were wide varieties of participant sample sizes (<10 up to hundreds) and study durations (<2 weeks up to a year). The metrics evaluated by technology, largely using inertial measurement units in wearable devices, included gait, tremor, physical activity, bradykinesia, dyskinesia and motor fluctuations, posture, falls, typing, sleep and activities of daily living.

**Conclusions:**

Home-based free-living testing in PD is being conducted by multiple groups with diverse approaches, focussing mainly on motor symptoms and sleep.

## INTRODUCTION

Parkinson’s disease (PD) is a chronic, progressive, disabling disorder of the central nervous system with a wide heterogeneity of clinical presentations and rates of progression. It is the second most common neurodegenerative disease, with the Global Burden of Disease Study [1] estimating a worldwide prevalence of around 6.1 million people.

PD is characterised by a large variety of motor symptoms (including bradykinesia, rigidity, tremor and dyskinesia) and non-motor symptoms (including sleep disturbance, cognitive impairment, genitourinary dysfunction, fatigue and pain) [2]. PD is a complex disease exerting a large burden upon patients and their carers, and financially upon healthcare systems [3].

### Issues with clinical rating scales

The MDS-UPDRS (Movement Disorder Society-Sponsored Revision of the Unified Parkinson’s Disease Rating Scale) [4] is the current gold-standard way of measuring disease severity in PD. It is a revised version of its predecessor clinical rating scale, simply called the UPDRS. The MDS-UPDRS has four parts, numbered I (with sub-sections A and B), II, III and IV, looking at non-motor and motor experiences of daily living, motor examination and motor complications. Parts IB and II are completed by the patient; otherwise the scale is completed by the rater. Sections or sub-sections of the MDS-UPDRS are frequently used in trials as primary outcome measures. The MDS-UPDRS is a valid [5] tool to measure a wide variety of different aspects of PD. However, it is also arguably a subjective [6], non-linear [7] scale which is biased toward certain aspects of the condition and which displays a ‘floor effect’ which renders it insensitive to early-stage disease [8]. In addition, the inter-rater variability of the MDS-UPDRS part III is up to 16 points [6]—to illustrate how substantial this variability is, it is notable that a change in UPDRS motor scores of 11 points in one person given a novel therapy in a clinical trial would represent a large clinically important difference (CID) and of about 3 points a minimal CID [9]. The MDS-UPDRS is predominantly used for clinical trials comparing therapeutic effects between groups on treatment versus placebo, to track longitudinal changes in PD cohorts over time or to guide clinical decision-making at the individual level in specific circumstances. However, the scale is not validated to permit comment upon what constitutes a clinically important difference (CID) in a single measured individual, given the test-retest variability at the single patient level is high. Similarly, scores comparing between small groups of individuals are also problematic and this, in-part, underlies the need for better ways of measuring outcomes. This is in order that true variability in symptoms, both intra-patient and inter-patient, can be measured with validity and accuracy. Moreover, the nature of the observed testing situation that the MDS-UDPRS requires means that confounds of observer bias and the ‘Hawthorn Effect’ (where aspects of performance are modified because the person is being observed) are introduced [10]. Finally, the single or episodically repeated ‘snap-shot’ of the person’s symptoms and clinical signs is perhaps under-evaluating the symptomatology of some patients with PD, which can fluctuate from day to day and hour to hour. Nevertheless, it remains the gold standard against which all other measures are compared, including sensor-based assessments.

### Technology in PD

There is recognition within parts of the PD academic community, voiced by the International Parkinson and Movement Disorders Society Task Force on Technology, that technology could provide a rich source of granular data which captures the disease and treatment-related fluctuations of this complex condition [11]. Technological sensors have the potential to continuously and unobtrusively measure aspects of PD, and the activities in the lives of those with PD, to give more reliable and objective evaluations of outcomes in PD.

The past 10–15 years has seen the emergence of a variety of technologies which have the potential to harness data to capture illness and health outcome measures, including smartphones [12, 13], wearable devices [11, 14], ‘smart homes’ (a home equipped with, e.g., lighting, heating, and electronic devices that can be controlled remotely by smartphone or computer) [15], advanced analytics and the Internet of Things (IoT) which is the interconnectivity via the internet between computing devices embedded in everyday objects [16].

Currently technology cannot replace may be able to assist the experienced clinician performing a detailed history and examination or a qualitative patient-reported experience of the disease. There are some symptoms which may prove extremely challenging to quantify using technology, such as pain, fatigue and rigidity. However, technology provides an opportunity to find a more sensitive and reliable way of measuring an individual’s symptoms, which in turn could answer the need in clinical trials for improved ways of evaluating disease-modifying therapies [17]. In clinical practice, its use could be adjunctive to routine face-to-face patient-clinician interactions; it remains to be seen how much this would change management plan formulation although some reports are encouraging [18].

The advent of new technologies brings the threats which perhaps are inherent to the management of electronic data including data security. Close thought will need to be given with regards to considering the pathways towards regulatory approval for devices or platforms if these technologies are to be widely adopted in clinical trials or clinical practice [19].

### Testing in the home or home-like setting

Generally, testing of new technologies takes place either in structured laboratory or clinic environments and/or in the home or home-like setting, otherwise known as the ‘free-living’ or naturalistic environment. Data can be collected from new digital devices through participants’ scripted activities (undertaken in a pre-determined sequence according to a script) or unscripted activities (where the subject is able to freely choose their activities without being told to perform certain tasks). This review is interested in the data gathered from unscripted activities in free living; this data collection may be continuous or otherwise.

The choice of the home as a testing location to focus on for this review derives from perceiving the opportunities that this setting may bring to patients, clinicians and researchers. For people with PD, being tested at home could enable better appreciation of some activities of daily living (ADLs) which occur more naturally away from a clinic or lab environment [20], rare events such as falls [21], activities which impact upon wellbeing and quality of life [22] and outcomes such as sleep quality which are costly and logistically difficult to measure longitudinally in the clinic/lab. Technology deployed to the home could provide measurements to the clinician/researcher which would otherwise have required clinician time to obtain [23], have scalability to large numbers of people with PD remotely [24], and reduce the cost of clinic visit/clinical trial contacts [25]. An extrapolation is that the home setting could improve generalisability in outcome measure results, for example by increasing inclusivity towards those people living outside the radius of a clinical treatment unit. The aforementioned, however, are hoped for benefits and not yet of proven utility.

However, prior to employing technology-assisted in the home for longer-term use, hurdles include the proof that there will be comparability in detecting motor and other impairments when compared to in-clinic assessments [26], the documentation of acceptability of the technology in people with PD, careful measures surrounding data management and navigating the technology towards regulatory approval [19].

### What this review adds to existing literature

To our best knowledge, this is the first systematic review focussing on the evaluation of technology-assisted outcomes, using both wearable and non-wearable devices, from free living within the naturalistic environment in PD.

This systematic review aims to find out what is described in this area: what technologies are being used to measure which PD outcomes in what nature of ecological environment, and to give an idea of how the technologies are being clinimetrically tested and validated. We wish to highlight the areas where future development is needed in order to produce a fully-validated and clinically-relevant set of outcomes measurable in PD from a person’s own home, using minimally intrusive and continuous monitoring.

## METHODS

A systematic review of all articles published from January 2000 until May 2018 was conducted using the following information sources: Medline, Embase, PsycInfo, Cochrane Database of Systematic Reviews and Cochrane Central Register of Controlled Trials and Web of Science.

The population being studied is people with PD; the intervention is the use of technology/technological devices to measure activities in the home or home-like environment; there is no specific comparator; the outcomes being measured are symptoms of PD and also activities of daily living evaluated from the free-living of the participants. A review protocol is available at https://research-information.bris.ac.uk/en/publications; the systematic review is registered in the International Prospective Register of Systematic Reviews, Prospero, under the identification CRD42018095479.

A combination of MeSH (Medical Subject Headings) terms and keywords were used in the search strategy. The authors borrowed from the published search strategy for a previous systematic review [14] and added additional pertinent terms when devising their strategy. Three blocks of these search terms were introduced and connected in the search strategy: the first relating to the condition of PD, for example “Parkinsonian disorders,” “basal ganglia diseases,”; the second to capture the concept of technology terms related to PD assessment, for example “technology,” “sensor,” “machine learning,” “accelerometer”; the third was related to the home or a home-like environment as the setting of the study, for example “home,” “naturalistic,” “free living”. The full list of search terms is available in Supplementary Table 1.

**Fig.1:**
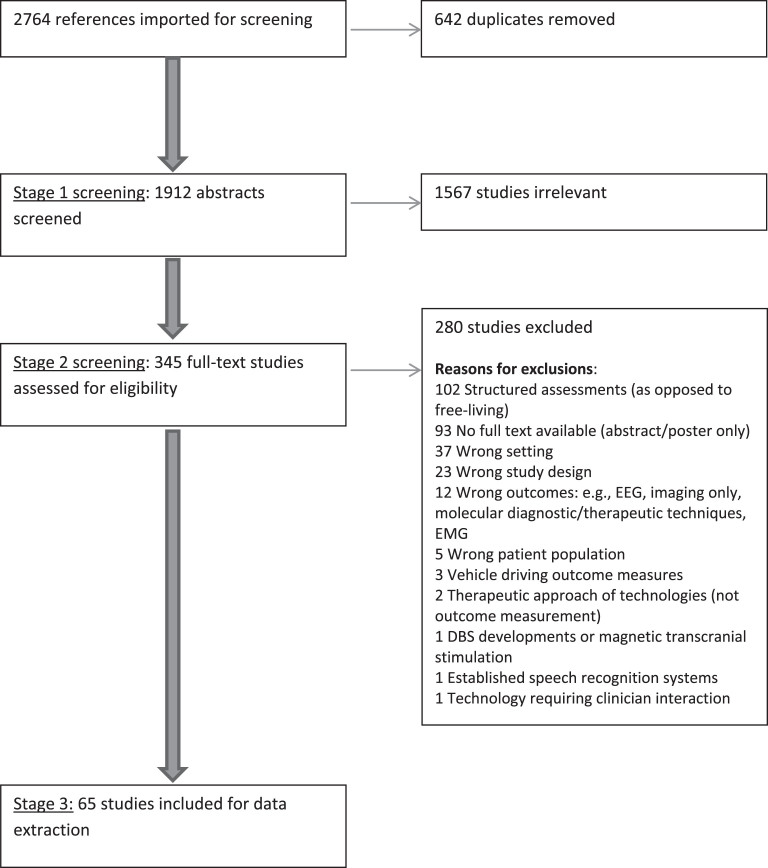
PRISMA (Preferred Reporting Items for Systematic Reviews and Meta-Analyses) flowchart.

The flowchart for study identification through to the studies included for data extraction, according to the PRISMA (Preferred Reporting Items for Systematic Reviews and Meta-Analyses) statement, is shown in Fig. 1. After identifying and de-duplicating the references from the sources mentioned above, the review was conducted in three stages:

Stage 1: Abstract screening

The studies’ abstracts were screened by C.M. with 10% of the abstracts randomly selected and screened by an independent reviewer, M.R., to check for accuracy. The criteria for the articles included in the next step were: the population of PD and the use of technology as part of the study.

Stage 2: Full-text screening and assessment to determine eligibility to be included in this review

All of the full-text studies were read and assessed for eligibility by C.M., 10% of these studies were selected at random and assessed by an independent reviewer, M.R, to check for accuracy.

The criteria to include a study in the next step of data extraction included: the study included a population of adults with a PD diagnosis; technology was used, for example (but not necessarily including) wearable and/or non-wearable devices, wireless/remote sensing technology, machine learning and/or algorithm development; the setting included a home-like environment or home environment, including environments where participants can move freely; outcome was the measuring, assessing or testing of any motor and non-motor aspect relevant to PD, as well as ADLs; the study included unrestricted/unscripted activities undertaken by participants.

Regarding the full-text studies, exclusion criteria for the next evaluation step were:

• The sole use of electromyography and/or electroencephalography (EEG) to generate outcomes;
• The sole use of questionnaires/scales;
• The sole use of technology requiring clinician interaction (e.g., remote consultations);
• The sole use of sphygmomanometers (i.e., blood pressure cuff with intermittent readings) to look at autonomic function;
• Genetic/Molecular diagnostic/therapeutic methods;
• Structured assessments in the home (as opposed to free living);
• Vehicle driving outcome measures;
• Imaging techniques only;
• Established speech recognition systems;
• New developments in deep brain stimulation (DBS);
• Magnetic transcranial stimulation only;
• Study design not involving testing of technology on participants at home (e.g., review, protocol, acceptability testing only);
• Articles not written in English language
• Therapeutic approaches of technologies only (as opposed to outcome measurement); and
• Full text was not available (through our University’s library access, and with cross-check using a mainstream widely-available search engine, Google Scholar).

The exclusion criteria were chosen to facilitate the inclusion only of studies which matched the review’s aims of evaluating the measurement of free living outcomes in PD in a free-living environment, specifically in the home as opposed to in a car for example.

A list of excluded manuscripts is provided as a supplement to this paper (Supplementary Table 2).

Stage 3: Data extraction from the included studies

Full evaluation of the included studies was completed by C.M., with independent evaluation by M.R. of 20% of randomly selected studies. Any mis-matches between the two reviewers were discussed face-to-face and resolutions were agreed by both parties.

Information on several pre-selected aspects was extracted using a customised table specifically designed for this review in Covidence systematic review software [28]. The aspects selected were intended to give the reader a grasp of the type of technologies being used, which aspects of PD were being measured and in what nature of location. This included the study design and any prior work to validate the technology in PD which appeared to have been carried out. To give an idea of the aspects of PD being evaluated, it was delineated whether motor, non-motor or ADLs (or a combination) were being measured, and whether single or multiple aspects/symptoms of PD were analysed. Of particular interest to this review is the location of technology testing—whether at home or in a home-like setting—and additionally the number of sensors (single or multiple) being used. The phrase ‘home-like setting’ is taken to mean a place which closely resembles a participant’s real home in terms of furniture/appliances where a participant can stay and move/live freely, but which is not in fact their true home.

Data was also extracted on each study’s inclusion and exclusion criteria, duration of home-based testing and the sample size used in each article.

Finally, details of the technologies used, and the outcomes generated, was collected. Largely, given that these studies were observational and exploratory in design, clinimetric properties such as repeatability and responsiveness to a pharmacological intervention were not expected to feature heavily in the studies’ design. However, any reported clinimetric properties (accuracy, responsiveness, reliability, test-retest agreement and agreement with the gold-standard clinical rating scale in PD) were extracted, whether those gathered from direct testing in the home/home-like environment in the studies or those documented in the manuscripts relating to previous testing.

## RESULTS

A total of 2764 studies were extracted by the search detailed above. After duplicates were removed, 1912 abstracts were screened by the authors. From these, 345 full texts were selected to be screened, of which 65 studies were included in this review.

An overview will be given about study designs used, the nature of the home/home-like environment in which the technologies were tested, the study sample size range and the duration of the studies. Subsequently, grouped by the metric(s) being measured, more detail about the outcome measurement, the nature of the technology used in measuring it, any validation undertaken and finally the documented clinimetric properties of the technology will be outlined.

### Study design

Of the 65 studies selected for data extraction, the study design was most frequently observational (57 out of 65, 88%). There were two papers detailing a case-control design whereby people with PD were directly compared to control participants when evaluating various parameters such as activity levels and keyboard typing metrics [29, 30]. In two studies, technologies were being used in the home environment as outcome measures in clinical therapeutic trials [31, 32] and a further four were randomised controlled trials or sub-studies thereof [33–36].

### Nature of home-like environment

Out of 65 studies, 61 described technology use in the participants’ own homes. When the participants’ actual home was not utilised, two studies described a ‘100-m^2^ laboratory arranged to simulate a home environment’ [37, 38] and two utilised a simulated ‘apartment/home environment’ [39, 40] as a test-bed.

### Sample size

The number of participants used to test technological systems in a naturalistic environment varied greatly between studies. Almost half of the studies (32) used between 10 and 49 participants in home testing of their technologies. However, a significant number used fewer than 10 or more than 100 participants (12 and 8 studies respectively). Table 1 gives details about the numbers of study participants.

**Table 1:** Sample size numbers indicating number of participants participating in free-living elements of selected studies

Sample size	Number of studies	Studies
Fewer than 10	12	Cereda et al.., 2010 [74], Das et al.., 2012 [68], Godfrey et al.., 2016 [62], Nakae et al.., 2011 [78], Pastorino et al.., 2013 [80], Perez-Lopez et al.., 2015 [81], Raknim et al.., 2016 [44], Stack et al.., 2016 [47], Vega et al.., 2016 [48], Wallace et al.., 2013 [32], Weiss et al.., 2011 [65], White et al.., 2007 [50]
10–49	32	Battista et al.., 2018 [66], Bayes et al.., 2018 [83], Bhidayasiri et al.., 2016 [31], Bhidayasiri et al.., 2017 [33], Cai et al.., 2017 [29], Cancela et al.., 2011 [51], Cancela et al.., 2014 [54], Cavanaugh et al.., 2012 [49], Cole et al.., 2010 [40], Cole et al.., 2014 [39], El Gohary et al.., 2010 [69], El Gohary et al.., 2014 [59], Fisher et al.., 2016 [85], Haertner et al.., 2018 [57], Hale et al., 2010 [75], Iluz et al., 2014 [58], Johansson et al., 2018 [42], Liddle et al., 2014 [43], Madrid-Navarro et al., 2018 [89], Mancini et al., 2015 [53], Ramsperger et al., 2016 [45], Rodriguez-Molinero et al., 2015 [84], Rodriguez-Molinero et al., 2018 [82], Roy et al., 2011 [38], Roy et al., 2013 [37], Sama et al., 2014 [56], Skidmore et al., 2008 [70], Sringean et al., 2016 [93], Sringean et al., 2017 [94], Tzallas et al., 2014 [55], Van Uem et al., 2018 [71], Van Wegen et al., 2018 [86]
50–99	13	Adams et al., 2017 [87], Arroyo-Gallego et al., 2018 [30], Wallen et al., 2015 [73], Cheng et al., 2017 [34], Del Din et al., 2016 [64], Gros et al., 2015 [90], Klingelhoefer et al., 2016 [91], Lloret et al., 2010 [76], Mancini et al., 2018 [60], Uchino et al., 2017 [95], Wallen et al., 2014 [72], Wallen et al., 2014 [36], White et al., 2009 [77]
100 or more	8	Cancela et al., 2013 [52], Cohen et al., 2016 [67], Del Din et al., 2017 [61], Silva de Lima et al., 2017 [41], Silva de Lima et al., 2018 [46], Lim et al., 2010 [35], Morris et al., 2017 [63], Prudon et al., 2014 [92]

### Duration of home study

Where the study duration was specified, the majority of the studies included in this review described testing of their sensors/technology over two weeks or less, however 10 studies detailed longer period of study time of up to a year [32, 34, 41–48]. Data from sensing technology was collected on more than one occasion by three studies. Cavanaugh et al. [49] used an ankle-worn wearable device to measure ambulatory activity levels. They compared the correlation between wearable sensor outcomes and clinical measures of gait and disease severity over a period of time (one year) by measuring ambulatory activity levels at the beginning and then again at the end of the year. Lim et al. [35] were investigating the impact of home cueing training on ambulatory activity monitoring outcomes over a period of 12 weeks at four specific times. White et al. [50] measured 24–48 hours of physical activity at 3 time points, separated by 7 days.

### Which PD outcomes were measured, by which technologies and how were these devices validated and clinimetrically tested?

The individual metrics (e.g., tremor, bradykinesia) have been described in their own sections below, however it is important to note that frequently several metrics were tested by technologies within the same paper, and therefore those papers are described in more than one of the sub-sections below.

### Gait

Gait, including freezing of gait, turning of gait and missteps, was evaluated by technologies in 19 studies included in this systematic review. Wearable technologies were used to measure this metric in 18 of the 19 papers, with the exception of Wallace et al. [32] who used in-depth video cameras to assess gait in the context of the impact of wearing strategically-weighted vests on this metric. Smartphones were included in the ‘wearable’ category given that they are worn on a person in a pocket or similar in order to capture the movements which inform gait analysis; these devices were utilised by two groups [34, 44]. Five of the studies outlined a multiple wearable device platform with which to measure gait [51–55], although four of these papers were from the same group (Cancela et al). Accelerometers were used in all the wearable devices detailed in the studies; gyroscopes were added to accelerometry in the technology described in ten papers [34, 51–59] and magnetometers were identified to also have been used in two of these studies [56, 57]. Validation of the technology outcomes measuring aspects of gait was referred to in 17 of the studies. These validation efforts, prior to the use of the technology in the free-living setting (either carried out as part of the studies included in this review themselves, or in previous clearly-referenced work), had taken place in laboratory-style settings where the activities were largely structured. Videotapes had been documented to have been used to provide ‘ground truth’ (real world accuracy provided by directing observing the activities; this can be achieved in a number of ways) to technology outcomes in 13 of the papers [39, 40, 51–58, 60–62], with instrumented walkways [61–64], motion analysis [53, 60], comparison with other similarly-positioned wearable devices [44] and direct researcher observation [58, 64, 65] also used to validate the technology. Clinimetric properties, detailed in Table 2, showed that 16 studies evaluating gait either evaluated clinimetric properties of their technologies themselves or referenced relevant other/previous work. Ten of those papers gave accuracy statistics, whilst seven looked at agreement between the technology-assisted outcomes and other tools like the UPDRS [51, 53, 60, 65], or an instrumented walkway [61, 63, 64].

**Table 2:** An outline of clinimetric property testing of the technologies as described by the studies included in this review

Study	Main outcomes measured	Device(s) used	Clinimetric properties
Adams 2017 [87]	Typing	Keystroke timing information analysis	This approach was able to discriminate between early-PD subjects and controls with 96% sensitivity, 97% specificity and an AUC of 0.98.
Arroyo-Gallego 2018 [30]	Typing	NeuroQWERTY algorithm	Sensitivity 0.73, specificity 0.69 in home setting (compared to controlled typing test at home).
			Agreement: significant moderate correlation with UPDRS III (correlation coefficient 0.34 in home setting)
Battista 2018 [66]	Tremor	1 tri-axial accelerometer at the waist	Sensitivity 99.3%, specificity 99.6%, accuracy 98.9%
Bayes 2018 [83]	ON/OFF fluctuations	Tri-axial accelerometer at wrist and smartphone app	Sensitivity 97%, specificity 88%
Bhidayasiri 2017 [33]	Sleep: nocturnal hypokinesia	Tri-axial accelerometer and gyroscope worn on trunk	Agreement: the change in numbers of turn in bed and degree of axial turn were mirrored by significantly greater improvements in clinical scale-based assessments, including the UPDRS total scores (*p* = 0.009), UPDRS-motor scores (*p* < 0.001),UPDRS-axial scores (*p* = 0.01), the Modified PDSS (*p* < 0.001), the Nocturnal Akinesia Dystonia and Cramp Scale (*p* = 0.003) and the eight-item PD Questionnaire (PDQ-8) scores (*p* = 0.01).
			Responsiveness: there was a significant difference, in favour of Rotigotine transdermal patch vs placebo patch, in change from baseline score in the number of turns in bed (*p* = 0.001), and degree of axial turn (*p* = 0.042).
Cai 2017 [29]	Physical activity	Accelerometer at wrist	Agreement: no correlation between wearable-recorded daily movement function and UPDRS-II and III scores.
			Responsiveness: activity level seemingly responsive to levodopa administration (One hour after taking levodopa, patients were significantly more active than 1 hour before the next dose (*p* < 0.05)).
Cancela 2011 [51]	Gait	Accelerometers on each limb, an accelerometer &gyroscope on the belt	Accuracy: the average error in the step frequency characterization was 1.88%.
			Agreement: There is not a direct correlation between variation in the magnitudes of signals measuring ON and OFF and variation in the UPDRS.
Cancela 2013 [52]	Bradykinesia, gait, dyskinesia, tremor	Accelerometers on each limb, an accelerometer &gyroscope on the belt	Accuracy of 93.73% for the classification of levodopa-induced dyskinesia severity, 86% of bradykinesia severity and 87 % for tremor (from previous work).
Cancela 2014 [54]	Tremor, bradykinesia, dyskinesia, gait parameters	1 accelerometer on each limb, an accelerometer + gyroscope on the belt	Previous work: 2 accelerometers can classify walking activity with 99% accuracy.
Cavanaugh 2012 [49]	Physical activity	Accelerometer at ankle	Agreement: ambulatory activity monitoring showed significant changes between baseline and the recording at 1 year in the amount and intensity of activity record (*p* < 0.007), however the clinical rating scales employed (UPDRS motor sub-section, 6-minute walk, UPDRS gait question 3.10) showed no significant change in this time period.
Cheng 2017 [34]	Gait and ambulatory activities	Accelerometer and gyroscope within smartphone	From previous paper [118]: The model was able to correctly distinguish gait activities (walking, stairs, jogging) from stationary activities (sitting, standing, lying down) with more than 98% of accuracy, Additional validation showed 96.9% accuracy for gait and 99.5% accuracy for balance.
Cohen 2016 [67]	Sleep activity, daytime physical activity, gait	Tri-axial accelerometer worn on wrist	The model accuracy for gait on a random validation set was 98.5% (precision 98.9%, recall 96%).
Cole 2010 [40]	Tremor, dyskinesia	Surface electromyographic sensors and tri-axial accelerometers (4 worn)	Tremor: sensitivity 93%, specificity 95%.
			Dyskinesia: sensitivity 91%, specificity 93%.
Cole 2014 [39]	Tremor, dyskinesia	Surface electromyographic sensors and tri-axial accelerometers (2 gathered data: shin and wrist)	Tremor:>95% sensitivity and specificity
			Dyskinesia:>90% sensitivity and specificity.
Das 2012 [68]	Tremor, dyskinesia	Tri-axial accelerometers, 5 devices, at wrists, ankles and waist	Accuracy 93% for tremor, 93% for dyskinesia.
Del Din 2016 [64]	Gait	Tri-axial accelerometer on lower back	Agreement (from a previous paper [119]): of the 14 gait characteristics compared with an instrumented walkway, agreement between instruments was excellent for four (ICCs 0.913–0.983); moderate for four (ICCs 0.508–0.766); and poor for six characteristics (ICCs 0.637-0.370).
Del Din 2017 [61]	Gait	Tri-axial accelerometer on lower back	Agreement (from previous paper [119]): of the 14 gait characteristics compared with an instrumented walkway, agreement between instruments was excellent for four (ICCs 0.913–0.983); moderate for four (ICCs 0.508–0.766); and poor for six characteristics (ICCs 0.637–0.370).
El Gohary 2014 [59]	Turning of gait	Tri-axial accelerometers, gyroscopes and magnetometers worn on lumbar spine	Accuracy: Compared to Motion Analysis and video, the algorithm maintained a sensitivity of 90% and 76% and a specificity of 75% and 65%, respectively.
Fisher 2016 [85]	Dyskinesia, ON/OFF fluctuations	Accelerometers worn on each wrist	In the clinical setting, specificity for dyskinesia detection was extremely high (0.99); high specificity was also demonstrated for home-derived data (0.93), but with low sensitivity (0.38). In both settings, sensitivity for on/off detection was sub-optimal.
			Agreement: ANN-derived values of the proportions of time in each disease state showed strong, significant correlations with patient-completed symptom diaries.
Godfrey 2016 [62]	Gait, falls	Tri-axial accelerometer, worn on back	1 fall correctly identified (only 1 PWP), and pre-fall event correctly segmented by algorithm, but 38 false positives (falls) also detected by algorithm.
			Agreement: correlated with fall in participant diary.
Gros 2015 [90]	Sleep: apnoea, hypopnoea, oxygen desaturation, pulse rate	Two respiratory inductance Plethysmography belts, a nasal pressure cannula and a pulse oximeter	Sensitivity of Portable Monitoring was 84.0%, 36.4%, and 50.0% for apnoea hypopnoea index cut-offs of 5/h, 15/h, and 30/h, respectively, using the same cut-offs on PM. Specificity was 66.7%, 83.3%, and 100%, respectively.
Hale 2008 [75]	Physical activity	Accelerometer, worn on central lower back	Reliability of the accelerometer measuring free-living physical activity: ICC 0.85; 95% confidence interval 0.74–0.91, *p* = 0.000).
			Test-retest reliability over 2 test periods, 7 days apart, showed intraclass correlation coefficient 0.81 (good), confidence intervals 0.29–0.96, *p* value 0.01.
Iluz 2014 [58]	Missteps	Tri-axial accelerometer/gyroscope on lower back	Accuracy: 93.1% hit ratio and 98.6% specificity.
Johansson 2018 [42]	Bradykinesia, dyskinesia	Tri-axial accelerometer, on wrist	Responsiveness: The UPDRS motor scores changed significantly (*p* < 0.05) but the wearable device’s summary scores (bradykinesia &dyskinesia) showed no significant change following dose adjustments of levodopa/carbidopa depending on the PKG’s measurements.
Klingelhoefer 2016 [91]	Sleep quantity and quality	Tri-axial accelerometer worn on wrist	Agreement: Both the duration of sleep and the duration of wakefulness in the PD-EDS (excessive daytime sleepiness) group (but not in the PD-NS (non-sleepy) group), measured by the accelerometer, correlated significantly on a high level with the total burden of non-motor symptoms (NMS) of PD as measured by NMSQuest as well as the overall sleep disturbance as measured by PDSS (*p* < 0.01).
Lim 2010 [35]	Physical activity	Accelerometers, 1 on each thigh and 3 on lower sternum	Reliability for monitoring gait performance:
			Fair to good reliability (ICC = 0.50–0.72) for registration time, static activities, sitting and standing and excellent reliability (ICC = 0.76–0.81) for dynamic activity, Walking > 5 seconds and Walking > 10 seconds.
			Responsiveness: Cueing training for 3 weeks produced significant improvements in activity monitoring: significant improvements were found for dynamic activity (*β*= 4.46; *p* < 0.01), static activity (*β*= –3.34; *p* < 0.01), walking (*β*= 4.23; *p* < 0.01), Walking periods > 5 seconds (*β*= 2.63; *p* < 0.05), and Walking periods > 10 seconds (*β*= 2.90; *p* < 0.01). All intervention effects declined significantly at 6 weeks follow-up.
Lloret 2010 [76]	Physical activity	Tri-axial accelerometers, worn on chest and thigh	Agreement: In non-dyskinetic patients, mean activity correlated moderately with UPDRS II scores (R = –0.21, *p* < 0.05)
			Responsiveness: No differences in activity level between ON state and OFF state during the acute levodopa challenge in the laboratory setting (*p* < 0.5) or the 72-hr ambulatory period were found.
Madrid-Navarro 2018 [89]	Motor (acceleration and time in movement); non-motor (sleep, skin temperature rhythms, light exposure)	Tri-axial accelerometer, skin temperature, wrist posture, light exposure, worn on non-dominant wrist	Accuracy: predictive accuracy of the A/T ratio proposed was 100% (Acceleration during the daytime (as indicative of motor impairment), time in movement during sleep (representative of fragmented sleep) and their ratio (A/T)).
			Agreement: no significant correlations were found between A/T and PD rating scales or subscales (UPDRS, *ρ*= 0.157, *p* = 0.62; UPDRS II. *ρ*= –0.19, *p* = 0.55; UPDRS III, *ρ*= 0.41, *p* = 0.19; UPDRS IV, *ρ*= –0.34, *p* = 0.28) and sleep quality scores (PDSS), *ρ*= 0.12, *p* = 0.71; PSQI, *ρ*= –0.28, *p* = 0.37). However, statistically significant negative relationships were found between acceleration during daytime (M10 V) and sleep quality scales (PDSS-2, *ρ*= –0.71, *p* = 0.008; PSQI, *ρ*= –0.74, *p* = 0.006).
			Responsiveness: the same patient was recorded three times throughout the course of the study. A 61-year-old woman with advanced PD was monitored before, 1 week after, and 6 months after starting intra-jejunal infusion of LCIG, (Levodopa-Carbidopa Intestinal Gel) an advanced therapy to ameliorate her motor symptoms. The A/T ratio increased from 0.15 to 0.75 and 1.99, 1 week and 6 months after the onset of treatment, respectively.
Mancini 2015 [53]	Turning of gait	Tri-axial accelerometers and gyroscopes, worn on lower back and one on each foot	Accuracy: Compared to Motion Analysis, the algorithm maintained a sensitivity of 0.90 and a specificity of 0.75 for detecting turns (found in a previous study).
			Agreement: The coefficient of variation of turn velocity showed a high correlation with the UPDRS motor score (r = 0.79, *p* = 0.01). Similarly, the correlation between the number of steps per turn (r = 0.61 and *p* = 0.03) and turn velocity (r = 0.61, *p* = 0.03) with the UPDRS motor score were statistically significant.
Mancini 2018 [60]	Turning of gait (and freezing of gait), periods of walking	Tri-axial accelerometer and gyroscope worn on lower back	Accuracy: Compared to Motion Analysis, the algorithm maintained a sensitivity of 0.90 and a specificity of 0.75 for detecting turns (found in a previous study).
			Agreement: Measures of the quantity and quality of turning, except for mean turn angle, were significantly associated with disease severity, as measured by the MDS-UPDRS Part III (ON medication). The variability of all the quality turning measures and turn angle were associated with gait speed, as measured in the lab in the ON state.
Morris 2017 [63]	Gait	Tri-axial accelerometer	Agreement: body-worn monitor gait model remained stable in free-living conditions compared to previously published model based on GaitRite data [119].
Nakae 2011 [78]	Physical activity	Tri-axial accelerometer, worn at waist	Agreement: positive correlations of specific metrics with rating scale choices (Functional Balance Scale, Functional Independence Measure (FIM), Parkinson’s Disease Rating Scale (PDRS), frequency of falls, Modified Falls Efficiency Scale, Functional Reach Test (FRT)), e.g., frequency of standing up from a chair significantly correlated with FIM (r = 0.729), PDRS (r = –0.639), gait velocity (r = 0.825), FRT (r = 0.732), and FEBS (r = 0.707). See paper for full detail of correlations.
Pastorino 2013 [80]	Akinesia	PERFORM wearable system: 4 tri-axial accelerometers, one on each limb and 1 accelerometer/gyroscope on belt	Agreement: good correspondence (88.2 ± 3.7 %) was observed (compared to patient diaries).
Perez-Lopez 2015 [81]	Bradykinesia, dyskinesia (combined to measure motor fluctuations)	Tri-axial accelerometer, worn on waist	Accuracy: Results are a mean sensitivity of 99.9% and a mean specificity of 99.9%.
Raknim 2016 [44]	Gait	Accelerometer in smartphone	Accuracy of step length estimation was about 98.3% (standard deviation 1.3%). Identifying changes in walking pattern in PWP: 94% accuracy.
Ramsperger 2016 [45]	Dyskinesia	Tri-axial accelerometers and gyroscopes, worn at ankle	Sensitivity 85%, specificity 98%, accuracy 0.96 for detection of dyskinesia.
			The wearable showed a correlation level of 0.61 (*p* < 0.001) with the clinical severity score (UPDRS dyskinesia score).
			In the home-based sub-study, all patients could be correctly classified regarding the presence or absence of leg dyskinesia.
Rodriguez-Molinero 2015 [84]	Motor fluctuations (measuring motion fluency)	Accelerometer, worn on waist	Sensitivity 0.91, specificity 0.90.
Rodriguez-Molinero 2018 [82]	Bradykinesia, dyskinesia, motor fluctuations	Tri-axial accelerometer, worn on waist	Accuracy: The positive predictive value of the algorithm to detect Off-periods, as compared with the patients’ records, was 92% (95% CI 87.33–97.3%) and the negative predictive value was 94% (95% CI 90.71–97.1%); the overall classification accuracy was 92.20%.
Roy 2011 [38]	Tremor, dyskinesia	Tri-axial accelerometer &surface electromyograph, 4 devices, worn on distal portion of each limb	The sensitivities/specificities for different severities of tremor and dyskinesia (mild, moderate and severe for each symptom) were between 91.9–99.3%.
Roy 2013 [37]	Tremor, dyskinesia	Tri-axial accelerometer &surface electromyograph, 4 devices, worn on distal portion of each limb	Tremor: sensitivity 90.2%, specificity 92.9%
			Dyskinesia: sensitivity 91.7%, specificity 89.5%.
Silva de Lima 2018 [46]	Physical activity	Accelerometer at wrist	Accuracy: gait detection algorithm accuracy was 98.5% (precision 98.9%, recall 96%) on the training data.
Skidmore 2008 [70]	Physical activity	Accelerometer worn above right lateral malleolus	When calibrated to the individual, accuracy > 95%.
			Agreement: Good correlations between measurements of number of steps taken per day &maximal activity levels and the UPDRS total score, the activity of daily living subscale, and the UPDRS motor function subscale, on and off medication, all *p* < 0.01.
Sringean 2016 [93]	Sleep: nocturnal hypokinesia	Tri-axial accelerometer and gyroscope, worn on both wrists, both ankles and trunk	Agreement: The interpretation of correlation coefficients indicated that there was a moderate correlation between duration of rolling over and UPDRS axial score (r = 0.619, *p* = 0.005) as well as NADCS score (r = 0.68, *p* = 0.001), and its akinesia sub-score (r = 0.46, *p* = 0.047). Similar correlations were observed between degree, velocity, acceleration, and item 28 of UPDRS on posture (r = 0.447, *p* = 0.039; r = 0.666, *p* = 0.002; r = 0.617, *p* = 0.005 respectively). Moderate and significant correlations were demonstrated between the episodes of getting out of bed (nocturia), and UPDRS-III (r = 0.579, *p* = 0.009) as well as UPDRS axial score (r = 0.498, *p* = 0.03), total LED (levodopa equivalent dose) (r = 0.475, *p* = 0.04), and night-time LED (r = 0.554, *p* = 0.014). The number of leg movements on the predominantly affected side also correlated with RBD1Q (r = 0.472, *p* = 0.041), the total NADCS (r = 0.493, *p* = 0.032), and its cramp sub-score (r = 0.475, *p* = 0.04).
Sringean 2017 [94]	Sleep: nocturnal hypokinesia	Tri-axial accelerometer and gyroscope, worn on both wrists, both ankles and trunk	Agreement: significant correlations were observed between the duration of supine position and the followings, including the UPDRS axial score (r = 0.482, *p* = 0.012), and the degrees of turns in bed (r = –0.745, *p* = 0.012).
Tzallas 2014 [55]	Tremor, bradykinesia, freezing of gait, dyskinesia	PERFORM wearable system: 4 tri-axial accelerometers, one on each limb, &1 accelerometer +gyroscope on belt	Tremor: 87% classification accuracy, 0.088 mean absolute error
			Dyskinesia: 85.4% classification accuracy, 0.31 mean absolute error
			Bradykinesia: 74.5% classification accuracy, 0.25 mean absolute error
			Freezing of gait: 79% classification accuracy, 0.79 mean absolute error.
Uchino 2017 [95]	Sleep: nocturnal kinetic parameters	Tri-axial accelerometer worn on abdomen	Agreement: Number of turnover movements in bed correlated negatively with disease duration (r = –0.305; *p* < 0.05), Levodopa-equivalent dose (r = –0.281; *p* < 0.05), Hoehn &Yahr staging (r = –0.336; *p* < 0.01), total score of UPDRS (r = –0.386; *p* < 0.01) and positively with Barthel-Index score (r = 0.365; *p* < 0.01).
Van Wegen 2018 [86]	Posture	Tri-axial accelerometer worn over xiphoid process of sternum	Significant decrease (average 5.4) degrees in trunk angle from baseline period (1 week) to intervention period with the vibration cueing device (1 week). It remains to be determined whether the corrected posture of 5.4 degrees is clinically relevant.
Wallace 2013 [32]	Gait	In-depth video camera	Responsiveness: Many of the entropy, asymmetry and peak-to-peak motion metrics showed statistically significant change with weighted vest intervention in the 4 subjects tested.
Wallen 2014 [72]	Physical activity	Tri-axial accelerometer, worn at waist	Accuracy: Manually counted steps from the video-recordings (mean steps = 339±34) compared to step counts produced by the accelerometer with each filter setting (Normal Filter mean step counts = 328±45, t(df = 14) = –2.07, *p* = 0.06; Low Frequency Extension Filter mean step counts = 345±22, t(df = 14) = 0.92, *p* = 0.37).
Wallen 2014 [36]	Physical activity	Tri-axial accelerometer, worn at waist	Agreement: Pedometer steps were significantly lower than accelerometer steps in the PD group (*p* = 0.002), Bland-Altman plots demonstrated wide limits of agreement between the instruments (pedometer and accelerometer).
Weiss 2011 [65]	Gait	Tri-axial accelerometer, worn on lower back	Agreement: While off medications, there was a significant correlation between UPDRS-Gait5 and average stride time (r = 0.50; *p* < 0.03) and between UPDRS-Gait5 and dominant-frequency (r = –0.50; *p* < 0.02).
White 2007 [50]	Physical activity	Activity monitor: two uni-axial and 1 bi-axial accelerometers used as sensors; sensors worn over both thighs and on sternum	Repeatability: The ICCs for the 7- and 14-day intervals ranged from 0.45 to 0.96, with walking-related measures showing the highest ICCs (range = 0.81 to 0.96). Across the three 24-hour periods (sessions 1 and 2, and the first 24 hours of session 3), the ICCs for walking-related measures were again high ranging from 0.87 to 0.92, i.e., the highest test-retest reliability for activities across 7- and 14-day intervals were found for walking-related measures in individuals with PD, indicating these measures have the highest stability compared to the other measures of functional activity.
White 2009 [77]	Physical activity	Activity monitor: two uni-axial and 1 bi-axial accelerometers used as sensors; sensors worn over both thighs and on sternum	Responsiveness: Higher doses of interdisciplinary rehabilitation (4.5 hours per week for 6 weeks) resulted in significant improvements in AM (activity monitor) measures for subjects with high baseline walking activity (p 0.02).

AUC, area under the curve; ICC, intraclass correlation coefficient; t, *t*-test; df, degrees of freedom; UPDRS-Gait5, sum of the UPDRS items that evaluated falling, freezing of gait, walking, postural stability, and gait; UPDRS, Unified Parkinson’s Disease Rating Scale; PDSS, Parkinson’s Disease Sleep Score; PSQI, Pittsburgh Sleep Quality Index; NADCS, Nocturnal Akinesia Dystonia and Cramp Score.

### Tremor

Tremor was measured in 13 studies (either as a single metric or combined with other metrics). All of these studies employed wearable devices, with single devices being used in four studies [41, 56, 66, 67], and multiple devices employed in the other papers [37–40, 52, 54, 55, 68, 69]. All researchers measuring tremor used accelerometers within the wearable devices, with five additionally using gyroscopes [52, 54–56, 69] and four studies using surface electromyographic sensors [37–40]. Clinimetric properties were mentioned in nine studies. These clinimetric properties are shown in Table 2; they comprise of statistics of accuracy and/or sensitivity/specificity, with all outcomes documented at > 90% apart from the classification accuracy for tremor of 87% produced by the PERFORM system (described in two papers [52, 55]). Validation efforts for the tremor metric were described by three groups. Cole et al. [40] validated algorithm development through video annotation of the PD metrics (tremor and dyskinesia) and used this validation to inform subsequent work [39]; Sama et al. [56] also used videotape comparison to validate. Silva de Lima et al. [41] described validation data from previous work using direct clinician-measured PD symptom severity [67].

### Physical activity

Of the studies included in the review, 17 measured the metric of physical activity. 13 of these papers described the use of single wearable devices [29, 36, 41, 46, 49, 67, 70–76], whereas four used multiple wearables [35, 50, 77, 78]. Each study used accelerometry only to measure physical activity except for Cereda et al, who used accelerometry, body temperature, skin conductivity and the sleep-wake rhythm to measure total daily energy expenditures, physical activity, number of steps and metabolic rate of their participants [74] in the context of investigating the impact of a low protein diet on physical activity and energy expenditure. Clinimetric properties were evaluated in 12 papers, with results detailed in Table 2. Accuracy of the technology was evaluated by Silva de Lima et al. [46], Skidmore et al. [70] and Wallen et al. [72] with > 95% accuracy found in the former two studies. Agreement with a number of different tools (including UPDRS II & III, Functional Independence Scale, Functional Balance Measure) was investigated by 5 papers [29, 36, 49, 70, 78] with varying results. These included the absence of agreement being noted, for example between accelerometry measuring physical activity and the UPDRS motor subscale (III) [29, 49]. Repeatability was looked at by White et al. [50] and Hale et al. [75] with test-retest evaluation which demonstrated good intra-class correlation coefficients in both papers. Responsiveness was evaluated by three studies: Lim et al. [35] looked at cueing training (significant improvements seen which declined at further follow-up), Lloret et al. [76] performed a levodopa challenge in the laboratory setting (no responsiveness of technology-assisted outcomes) and White et al. [77] noted the responsiveness of their accelerometry data to inter-disciplinary rehabilitation (improvements seen in group with high baseline walking activity). Validation work was referred to in support of seven papers’ findings. Silva de Lima et al. looked at gait detection and activity level and used clinician-observed structured assessments to produce a labelled training dataset [67] with which to inform their subsequent work included in this review [41, 46]. White et al. [50, 77] referred to a previous study using videotape to validate activity monitoring [79] and Lim et al. [35] also referred to this study’s validity testing in their paper. Wallen et al. [72, 73] used videotape to record a structured 3-minute walk in 15 people with PD to aid validation of their technology-assisted outcome measures [72]. Cavanaugh et al. compared the monitor’s step counts identified via a flashing indicator light with visual observation [49].

### Bradykinesia

Often combined with other motor outcomes such as dyskinesia or gait, eight studies investigated the ability of technology to measure bradykinesia/akinesia in their participants [42, 52, 54–56, 80–82]. All the research groups used wearable devices to measure bradykinesia/akinesia: four used single devices whereas four used multiple wearable devices. All of the devices employed to measure bradykinesia/akinesia contained accelerometers, with gyroscopes also in four devices [52, 54, 55, 80] and a gyroscope/magnetometer within one [56]. Validation was attempted through the use of videotape [55, 56], comparison with the UPDRS [42, 52], the use of telephone calls from researchers and comparison with participant study diaries [82]. Clinimetric properties were evaluated in six studies, and included measurements of accuracy [52, 55, 81, 82] and agreement (Pastorino et al. [80] documented good correspondence of 88.2% between technological outcomes and participant diaries). Responsiveness to dose adjustments of levodopa/carbidopa was assessed in a study by Johansson et al. [42], with the UPDRS motor scores changing significantly (P < 0.05) but the wearable device’s summary scores (bradykinesia & dyskinesia) showing no significant change following dose adjustments of levodopa/carbidopa. More detail about clinimetric properties is in Table 2.

### Dyskinesia and motor fluctuations

Motor fluctuations specifically were evaluated as a stand-alone outcome measure by two studies [83, 84], and they were evaluated alongside the dyskinesia outcome measure in three papers [81, 82, 85]. Dyskinesia was measured by technologies in 11 further papers included in this review [37–40, 42, 45, 52, 54–56, 68]. Wearable devices were used in all studies which evaluated dyskinesia and/or motor fluctuations, with seven using single wearable devices. Sama et al. [56], Rodriguez-Molinero et al. [82, 84] and Perez-Lopez et al. [81] used devices located at the waist or on the belt, Johansson et al. [42] and Bayes et al. [83] used a device at the wrist and Ramsperger et al. [45] used an ankle-worn device. Accelerometers were ubiquitously employed; they were used in some studies without other sensing technology and in other papers alongside gyroscopes [45, 52, 54–56], magnetometers [56] and surface electromyographs [37–40]. Validation of dyskinesia outcome measurements had been documented in eight papers, with various comparators used as ground truth to aid validation, including videotape recordings [39, 40, 55, 56, 84], direct clinician evaluation/observation [42, 83, 84], and participant diaries plus telephone calls from a researcher [82]. The clinimetric properties of the technologies under evaluation, where discovered, are detailed more fully in Table 2. The authors of this review were able to find some clinimetric evaluation in 14 of the papers: accuracy/sensitivity/specificity were the most frequently mentioned properties [37–40, 45, 52, 55, 68, 81–85]; agreement with the UPDRS dyskinesia score [45] and participant diaries [85] was also noted by specific studies; the responsiveness results from Johansson et al. [42] are noted in the sub-section above (bradykinesia).

### Posture

A single study looked exclusively at posture in the context of investigating whether posture detection and subsequent vibrotactile trunk ankle feedback could improve posture of participants [86]. The single tri-axial accelerometer-containing device located over the xiphoid process of the sternum was used both to collect movement data and provide feedback to the participant. Validation testing was not immediately evident, however responsiveness of the device’s measurement of posture as a result of the intervention showed a significant decrease (average 5.4 degrees) in trunk angle from baseline period to intervention period with the vibration cueing.

### Falls

Godfrey et al. [62] looked at the identification of falls in the free-living environment using a single wearable tri-axial accelerometer located on the back. The ground truth was provided by the participant diary recording the single fall which occurred during the study; this fall was correctly identified by the wearable device and the pre-fall event was correctly segmented by the algorithm, however 38 false positives (falls) were also detected by the algorithm. Stack et al. [47] used wearable devices (tri-axial accelerometers and gyroscopes worn on each wrist, each ankle and lower back), along with the non-wearable Kinect camera, to evaluate movement around the house in order to identify high risk of falling in their participants.

### Typing

The analysis of the typing patterns to investigate motor impairments in PD was conducted by three studies. Adams et al. [87] used keystroke timing information analysed by machine learning classification models to successfully discriminate between early-PD subjects and controls with a 96% sensitivity, a 97% specificity and an area under the curve (AUC) of 0.98. Arroyo-Gallego et al. [30] employed a ‘NeuroQWERTY’ algorithm and this had a sensitivity of 73% and specificity of 69% in home-based testing when compared to a controlled typing test in this environment. There was significant moderate correlation with the UPDRS III with correlation coefficients of 0.50 in clinic and 0.34 at home. Prior validation work of this algorithm, comparing typing outcomes of people with PD vs controls and comparing with the UPDRS III, had been conducted in a laboratory setting [88]. A third study by Vega [48] employed all of the sensors and interfaces within a smartphone, complemented by ambient, spatial and other web data sources, to analyse various behaviours including typing patterns, social and phone usage patterns and motor activities [48]. They propose that these metrics could signify hand movement performance and patterns; however the study detailed early pilot work at this stage which had not been clinimetrically tested or validated.

### Sleep

Sleep had been measured in 11 of the studies included in this systematic review [31, 33, 41, 67, 89–95]. Wearable devices were used to measure sleep outcomes in all of these studies. Single devices were used in eight of the 11 studies, worn on the trunk, wrist [41, 67, 89, 91], lower limb [92] and abdomen [95]. The location of the technology depended on what aspect of sleep was being evaluated, for example Prudon et al. using lower limb placement to measure periodic limb movements of sleep [92]. Multiple device platforms were described in three of the studies [90, 93, 94]; of note, Gros et al. [90] were interested in quantifying apnoea, hypopnoea, oxygen desaturation and pulse rate using two respiratory inductance plethysmography belts, a nasal pressure cannula and a pulse oximeter. Accelerometers were used in all of the wearable devices, accompanied by gyroscopes in four papers (all using the ‘NIGHT-Recorder’ device to measure nocturnal hypokinesia) [31, 33, 93, 94]. Validation had been attempted, if documented, in five studies, with the use of sleep diaries [31, 33, 93, 94], polysomnography [92] and night-time video recordings [94] to provide ground truth. Clinimetric properties are detailed in Table 2; however, there was a range of information about accuracy and agreement with clinical rating scales and questionnaires. Two studies looked at responsiveness of the technology-assisted outcome measures, one in the context of Rotigotine treatment [33] (where the Rotigotine was shown by the technology to effect a significant difference in change from baseline score in the number of turns in bed (*p* = 0.001), and degree of axial turn (*p* = 0.042)) and the other in response to Levodopa/Carbidopa Intestinal Gel treatment [89]. In this particular study by Madrid-Navarro et al, the same patient was recorded three times throughout the course of the study. She was a 61-year-old woman with advanced PD who was monitored before, 1 week after, and 6 months after starting intra-jejunal infusion of Levodopa-Carbidopa Intestinal Gel by a single tri-axial accelerometer with additional skin temperature, wrist posture and light exposure measurements worn on the non-dominant wrist. The A/T ratio (the ratio of acceleration during the daytime over time in movement during sleep) increased from 0.15 to 0.75 and 1.99, 1 week and 6 months after the onset of treatment, respectively.

### Activities of daily living

Vega [48], described above in context of evaluating typing, used the sensors and interfaces within a smartphone to identify latent behavioural variables which he could use to measure outcomes in PD. These variables included daily living activities such as phone usage patterns, social patterns and indoors routine. Given the early nature of this pilot work there was no validation/clinimetric testing documented. The metric of ‘lifespace’, a phrase given to the number of trips outside a home of > 500 metres, the length of distance travelled and approximate location to which travelled, was measured by Liddle et al. [43] using a single Global Positioning System within an Android smartphone. No validation or clinimetric testing was mentioned.

## DISCUSSION

This systematic review is, to our best knowledge, the first to evaluate the existing evidence around the use of both wearable and non-wearable technology which passively evaluates unstructured activities (free-living) in the home or home-like environment in order to generate outcome measures in PD. It complements work looking at wearable technologies specifically [96–99], outcome measures in PD using technology in any testing environment (technologies are largely tested in a laboratory environment) [11, 14, 100] and more specific reviews looking at individual symptom assessment using sensors [101, 102]. Additionally, for those interested, this review provides some thoughts on future avenues to improve and expand this approach to PD outcome measure testing at home.

### Types of studies

This review found that of the 65 studies selected for data extraction, the study designs were mostly observational which is perhaps intuitive given the passive nature of free-living monitoring in which the authors were interested. The studies were set in the participants’ real home setting with only infrequent exceptions [37–40]. Home-based testing may be beneficial in PD partly to cater for those patients who are limited geographically or practically from attending frequent clinic assessments [103], partly to save costs [25] and importantly to achieve ecological validity in the assessment of PD symptoms which are poorly captured in ‘snap-shot’ reviews in a laboratory or clinic environment [8].

The sample sizes used ranged widely from fewer than 10 to several hundred participants. The ideal numbers of participants to include in such studies depend on the intent of the researchers: whilst 10–12 participants may be enough to create a training dataset [104], smaller studies cannot sufficiently reflect the heterogeneous spectrum of clinical PD [105], therefore larger studies will ultimately be needed in order to ensure that the technologies are generalizable. The large sample sizes displayed by a number of papers [35, 41, 46, 52, 61, 63, 67, 92] are good indicators of the encouraging potential of technology to be scalable for use in clinical care and in trials but the data required to guide power calculations for future investigations employing these types of outcome measures remains yet to be determined.

The long duration of 10 studies of between 2 weeks and 1 year is encouraging as it demonstrates the potential to employ technology for periods of time sufficient to capture rare events such as falls [106], which can be difficult to recreate for evaluation in a laboratory environment. Interestingly, we found technology-assisted outcome measures being used in an episodic way by some groups [35, 49, 50] instead of continuously. These ‘snap-shot’ measurements potentially lend themselves more towards adjunctive outcome measurements in clinical trial contacts or for use when a patient with PD visits the outpatient clinic.

### Technologies used

The most frequently investigated sensors for the monitoring of PD symptoms were inertial measurement units within wearable devices and/or smartphones. This is possibly because these devices are commercially available, low cost, easy to use, small and are very frequently used anyway by the general population, so are likely to be acceptable. The development of the Internet of Things has also made it easier to transfer large amounts of data from wearables to electronic storage without needing to plug the wearables in using extra hardware. The use of non-wearable devices within our included studies was limited to depth video cameras and computer keyboards.

We found a significant number of studies utilising multiple sensors, as opposed to a single sensor, to measure outcomes in PD. The term ‘multiple sensors’ could include multiple wearable sensors, a combination of wearable and non-wearable devices, or multiple non-wearable sensors. There is currently no consensus on the optimum number of sensors to use to measure outcomes in PD; the balance is between the system being unobtrusive and acceptable enough for deployment into a participant’s life for prolonged periods of time versus the need to avoid loss of potentially relevant information through using too few sensors. When considering acceptability of technology-observed measures in PD, the authors are not aware of any overwhelmingly negative reports; however publication bias favouring publication of positive acceptability outcomes of technology is a possibility in this circumstance. Due to the fact that wearable devices are not completely unobtrusive and will always carry some physical burden when compared to non-wearable devices, it is possible that alternative approaches will (continue to) find their way into research and clinical practice. For example, the concept of a ‘smart home’ (with inbuilt unobtrusive sensors) added to wearables, smartphones passively monitoring and the Internet of Things could provide researchers with a menu of valuable tools.

### What is being measured?

With respect to motor outcomes, we found that the devices are being used to measure outcomes such as gait, physical activity levels, bradykinesia or akinesia, dyskinesia and tremor, but there were no papers evaluating rigidity and few which were measuring falls or posture. Additionally, technology was used to measure motor fluctuations in five of the included papers [81–85] with some encouraging results in terms of accuracy (see Table 2). Motor fluctuations are a relevant burden for patients [107] but are arguably inadequately quantified by the gold-standard clinical rating scales frequently used in clinical trials and in clinical practice. This review demonstrates that this metric, and other motor outcomes, are starting to be measured in a continuous and unobtrusive way in the home setting although much further work in this area is required.

Considering non-motor symptoms, sleep is evaluated in 11 studies, predominantly using wearable devices. This patient-relevant [108] ‘outcome’ in PD is very complicated with various sleep diagnoses being more common in the PD population (e.g., REM sleep behaviour disorder, restless legs syndrome, insomnia) [109]. Other non-motor outcomes remain largely unmeasured in the home environment. This is important, since symptoms such as depression, cognitive dysfunction and autonomic function are known to impact on health-related quality of life and wellbeing [22, 71]. In one survey, almost half the respondents felt that non-motor symptoms had a greater impact on quality of life than motor symptoms [110]. The methodology of how to measure non-motor symptoms such as fatigue or pain using technology may appear challenging, however there is potentially interesting work linking wearable accelerometer data used to measure bradykinesia with constipation outcomes [111]. This provides the hope that technology can be used in conjunction with other tools to measure these symptoms in PD at home, the under-measurement of which is highlighted by this review.

Activities of daily living were quantified to an extent in two papers, although one described early pilot work so far only [48] and the other investigated a metric of ‘lifespace’ which involved trips from the home to other places and not activities within the home itself [43]. It has been shown that ADLs and the ability to perform them have a significant impact on health-related quality of life [112]. Currently, ADLs are often evaluated through clinician questions or in the MDS-UPDRS part II, which is performed normally in an outpatient setting on regular occasions, leaving weeks/months between assessments. Researchers have argued that the best location to assess everyday functional abilities is in a participant’s own (home) environment [113]. Our review highlights that the measurement of free-living ADLs at home is an avenue for future work and is relatively unexplored.

### Validation

A home-based sensor system, continuously monitoring the symptoms of PD, could be a pathway towards personalising medical treatments and reducing hospital visits both in clinical care and in clinical trials [96]. However, the validation of such a system in a participant’s home, remote from researchers who could annotate the activities and potentially without a video camera system to provide ground truth, presents challenges as described below.

There are various aspects to consider when discussing the validation of technology-assisted outcome measures in PD. A focus of validation can be on the technical aspects of the study, for example to validate whether an algorithm operating on the data gathered from sensor(s) is meeting a defined set of requirements. This is distinct from clinical validation where validation is a check that the technology-derived outcome is an accurate representation of the symptom/behaviour/body function being measured. Clinical validation can be achieved in a number of ways, often including the use of study participant diaries, direct observation by a researcher and/or annotation of video data to give a ground truth. A good quality validation dataset would depend on the symptom or activity under evaluation; generally, due to the complex and heterogeneous nature of the condition, having a dataset which incorporates many individuals would increase the generalisability of the results of validation [104]. A ground truth involving frequent annotations [114] from expert clinical raters (with which to compare to sensor data) should also improve the ability to accurately validate the technology outcomes.

This review found that 35 studies mentioned validation attempts of their technologies in PD using various methods: videotape analysis was used to validate technologies described by 19 papers [39, 50, 51, 53, 55–61, 67, 72, 73, 77, 84, 85, 94], direct clinician observation in six [41, 46, 49, 58, 65, 83], participant diaries in seven [31, 33, 62, 82, 85, 93, 94], comparison with the MDS-UPDRS or another clinical rating scale was used in three studies [42, 65, 80], an instrumented walkway in three [61, 63, 64], motion analysis in three [53, 59, 60], comparison to other wearable devices or device filter settings in three [36, 44, 72], telephone calls from a researcher in one [82], polysomnography in one paper [90] and sleep respiratory home monitoring (assessing snoring, nasal airflow, oxygen saturation, body position, and respiratory and abdominal wall movements) in another [92].

In terms of location of validation efforts: participant diaries, telephone calls from researchers and comparison with other wearable devices or device filter settings could all be collected in the home setting. The single paper using polysomnography performed this validation in the laboratory [90], but an attenuated sleep monitoring system was deployed to the participants’ home in another paper [92]. As far as the authors of this review could ascertain, the validation efforts using video recordings, motion analysis, instrumented walkways and direct clinician observations/interactions were all conducted in a laboratory setting (either in the paper itself or in previous work) prior to the deployment of the technology to the naturalistic setting.

The transfer of validated technology-assisted outcomes from laboratory to home settings is not without issues. These include the different contexts (environment and task) which could change movement outcomes and algorithm accuracies; the ability to prevent hardware damage/accidents for example is more limited; external factors such as mood and medication could introduce variability. Of interest, Fisher et al. [85] used an Artificial Neural Network to identify disease states (dyskinesia, ON/OFF state) first in the laboratory and then unsupervised at home, and compared to diaries and clinician rating of disease state. In the laboratory, the specificity for dyskinesia detection was 99%, and in the home environment the specificity was also 93%, but the sensitivity was low at 38% with suboptimal sensitivity for on/off state. This demonstrates that this technology which was validated in the laboratory was not ready for deployment to the home; this could potentially be the case for many such technologies if tested in a similar way.

Validation in the home environment itself, particularly when measuring unstructured/free-living activities, can be challenging: the presence of cameras or an observer could influence the behaviour of a study participant [10]; also the heterogeneity of the layout of peoples’ homes could make camera placement to achieve reliable ground truth difficult. The use of participant diaries to validate the technology, often with a diary entry frequency of every 30 minutes, arguably would also disrupt the free living being evaluated by technology and are also limited by self-reporting biases, cognitive impairments limiting recording accuracy and motor impairments which make symptom recording challenging [115]. The limitations of the MDS-UPDRS have already been discussed and lead to this clinical rating scale not being particularly helpful for most technology validation needs. As it currently stands, testing the technology against itself using test-retest repeatability and responsiveness, for example, may be the best way of validating results.

### Clinimetric properties

The diverse study designs, methods, sample sizes and statistical analyses of the papers included in this review mean that clinimetric properties could not be compared to each other. Twelve studies did not include detail about clinimetric properties tested to the best of the authors’ knowledge, however all other studies tested one, or a combination of, clinimetric properties including accuracy/sensitivity/specificity, agreement, repeatability and responsiveness. The discussion surrounding how successful each paper was in assessing clinimetric properties is beyond the scope of this paper as each study was conducted in a different manner with diverse patient populations, testing procedures, technologies and algorithms.

### Limitations

This systematic review has limitations. One author undertook most of the abstract and full text screening work (C.M.). However, 10% of these abstracts and full texts were cross-checked and screened by another author (M.R.) for accuracy. There were a number of papers for which full-texts could not be obtained from an initial university electronic library database search. In these instances the authors used further search and direct enquiry within a university library service for named manuscripts and also employed the use of a mainstream (academic) search engine; however if the full text still remained unavailable these studies were excluded and this exclusion could have impacted upon our study results. These excluded papers are listed in the Supplementary material (Table 2). Another limitation is the potential for publication bias and the potential that small positive studies were more likely to be published than those with negative results. The time frame for literature search from 2000 was a bridge between two excellent reviews detailing the previous 10 years’ literature on the topic [14, 96] and examples of other authors who conducted open-ended searches [11]. Relevant papers from pre-2000 may have been omitted, however one previous systematic review documented more than 50% of their studies included had been published in the preceding 3 years to 2016 [96], so the likelihood of substantial numbers of studies having been missed by our search strategy is low.

### Future directions

We found that most studies had investigated the use of technology-assisted outcome measures to look at motor features of PD or sleep. There is a need for a focus to be directed towards non-motor symptoms and ADLs, impairment of which significantly impacts upon the health-related quality of life of people with PD [112]. Since there are so many separate groups working across the world looking at similar technological sensors to measure outcomes in PD, it is worth noting that collaborative efforts including dataset sharing/cross-validation approaches will be vital to ensure that groups avoid replicating work that is already done and instead can move this exciting field forwards. Advances in IoT technology have brought with them privacy and data security concerns and it is important that these are addressed robustly to avoid data breaches and loss of confidence in what promises to be a valuable way of measuring PD.

Additionally, a vital issue is whether regulatory bodies (for example the Federal Drugs Agency or European Medicines Agency) recognise and approve the measurement systems in order to build for their futures as widely-accessed tools in clinical care and clinical trials. Most tools are not yet approved by the FDA/EMA. A further point is whether these devices have CE (Conformité Européene) marking to provide some assurance that they comply with the essential requirements of the relevant European health, safety and environmental product legislation, including enforcing legal obligations regarding data. The latter is particularly important and brings legal obligations regarding data privacy and ownership. Moreover, in order for the technology to be recommended by NICE (National Institute of Clinical Excellence) in the United Kingdom (UK) for example, enough clinical and economic evidence needs to be gathered that the device(s) will be beneficial to health and good value for money for the UK’s National Health Service [116].

There are constructive initiatives aiming to move this field forward (with industry, academics and others) in a collaborative and appropriate way towards the adoption of health technologies to measure outcomes in PD, such as the Movement Disorders Society Taskforce on Technology’s recent ‘roadmap’ [19] and Mobilise D [117].

### Conclusion

A number of groups are using technological devices to produce outcome measures in PD through the evaluation of free-living activities in the home or home-like environment. This will continue to be important as we learn how sensors can best be used to unobtrusively and accurately measure clinically and functionally important aspects of this common and debilitating disease. This review provides an overview of the current evidence in free-living home-based sensor testing in PD. It will be vital to continue to keep up-to-date with new advancements in this rapidly evolving scientific field.

## CONFLICT OF INTEREST

The authors have no conflict of interest to report.

## Supplementary Material

Supplementary Table 1

Supplementary Table 2
