# Supplementary material for: Systematic Review Looking at the Use of Technology to Measure Free-Living Symptom and Activity Outcomes in Parkinson’s Disease in the Home or a Home-like Environment
Source: J Parkinsons Dis. 2020 Apr 3;10(2):429–54. doi: 10.3233/JPD-191781 (PMC7242826; doi:10.3233/JPD-191781)
Supplement: Supplementary Table 1 [file jpd-10-jpd191781-s001.pdf]

Supplementary Material

Systematic Review Looking at the Use of Technology to Measure Free-Living Symptom and Activity Outcomes in Parkinson's Disease in the Home or a Home-like Environment

Supplementary Table 1. Literature Search Strategy for this review including all Search Terms according to the individual databases searched

**PsycINFO search**
(MAINSUBJECT.EXACT("Technology") OR ((diagnostic techniques) AND (MAINSUBJECT.EXACT("Mobile Devices") OR MAINSUBJECT.EXACT("Automated Speech Recognition") OR MAINSUBJECT.EXACT("Telemetry") OR MAINSUBJECT.EXACT("Actigraphy") OR MAINSUBJECT.EXACT("Telemedicine") OR MAINSUBJECT.EXACT("Spectroscopy") OR MAINSUBJECT.EXACT("Computer Assisted Diagnosis") OR MAINSUBJECT.EXACT("Biotechnology") OR MAINSUBJECT.EXACT("Computer Software") OR MAINSUBJECT.EXACT("Virtual Reality") OR MAINSUBJECT.EXACT("Computer Assisted Therapy") OR MAINSUBJECT.EXACT("Computer Applications") OR MAINSUBJECT.EXACT("Self-Evaluation") OR MAINSUBJECT.EXACT("Monitoring") OR MAINSUBJECT.EXACT("Machine Learning") OR MAINSUBJECT.EXACT("Cellular Phones") OR MAINSUBJECT.EXACT.EXPLODE("Artificial Intelligence"))) OR (sensor OR sensors OR nonwearab* OR "non-wearab*" OR webcam* OR "structured light" OR "artificial intelligence" OR assessment OR evaluation OR "cloud computing" OR telerehabilitat* OR "tele rehabilitat*" OR "machine learning" OR kinesis OR smartphone* OR "smart phone*" OR (mobile* NEAR/3 (technolog* OR device* OR phone*)) OR cellphone* OR "cell phone*" OR "speech recognition" OR software OR platform* OR accelerometer* OR gyroscope* OR magnetometer* OR actigraph* OR wearab* OR device* OR "big data" OR "internet of things" OR "closed loop" OR hybrid OR (home* NEAR/5 monitor*) OR quantitative OR algorithm* OR telemetr* OR instrumented OR "virtual reality" OR nonmobile OR portab* OR nonportab* OR technolog* OR (static NEAR/3 (technolog* OR device*))))
**AND**
((MAINSUBJECT.EXACT.EXPLODE("Parkinson's Disease") OR MAINSUBJECT.EXACT("Basal Ganglia") OR MAINSUBJECT.EXACT.EXPLODE("Parkinsonism")) OR not(parkinson*))
**AND**
(wearab* OR nonwearab* OR home OR homes OR homelike OR homebased OR naturalistic OR ecologic* OR "free living")
**AND**
pd(20000101-20191231)
**EMBASE search**
technology/ OR neurologic examination/ OR wireless communication/ OR remote sensing/ OR medical technology/ OR biomedical engineering/ OR telerehabilitation/ OR exp artificial intelligence/ OR orientation/ OR automatic speech recognition/ OR sound detection/ OR electrical equipment/ OR three dimensional imaging/ OR image processing/ OR outcome assessment/ OR symptom assessment/ OR self evaluation/ OR investigative procedures/ OR biomedical technology assessment/ OR medical informatics/ OR "point of care system"/ OR

machine learning/ OR mobile application/ OR mobile phone/ OR smartphone/ OR personal digital assistant/ OR software validation/ OR physiologic monitoring/ OR accelerometry/ OR actimetry/ OR ambulatory monitoring/ OR remote sensing/ OR telemetry/ OR virtual reality/ OR software/ OR thermography/ OR (sensor or sensors or nonwearab* or "non-wearab*" or webcam* or "structured light" or "artificial intelligence" or assessment or evaluation or "cloud computing" or telerehabilitat* or "tele rehabilitat*" or "machine learning" or kinesis or smartphone* or "smart phone*" or (mobile* adj3 (technolog* or device* or phone*)) or cellphone* or "cell phone*" or "speech recognition" or software or platform* or accelerometer* or gyroscope* or magnetometer* or actigraph* or wearab* or device* or "big data" or "internet of things" or "closed loop" or hybrid or (home* adj5 monitor*) or quantitative or algorithm* or telemetr* or instrumented or "virtual reality" or nonmobile or portab* or nonportab* or technolog* or (static adj3 (technolog* or device*))).mp. [mp=title, abstract, heading word, drug trade name, original title, device manufacturer, drug manufacturer, device trade name, keyword, floating subheading word, candidate term word]

AND

exp parkinsonism/ OR extrapyramidal syndrome/ OR parkinson*.mp.

AND

(wearab* or nonwearab* or home or homes or homelike or homebased or naturalistic or ecologic* or "free living").mp.

AND

limit to yr="2000 -Current"

Medline, Cochrane and CENTRAL search

(TECHNOLOGY/ OR "DIAGNOSTIC TECHNIQUES, NEUROLOGICAL"/ OR "WIRELESS TECHNOLOGY"/ OR "REMOTE SENSING TECHNOLOGY"/ OR "BIOMEDICAL TECHNOLOGY"/ OR "BIOMEDICAL ENGINEERING"/ OR TELEREHABILITATION/ OR exp "ARTIFICIAL INTELLIGENCE"/ OR KINESIS/ OR "SPEECH RECOGNITION SOFTWARE"/ OR "SOUND SPECTROGRAPHY"/ OR "ELECTRICAL EQUIPMENT AND SUPPLIES"/ OR "IMAGING, THREE-DIMENSIONAL"/ OR "IMAGE PROCESSING, COMPUTER-ASSISTED"/ OR "PATIENT OUTCOME ASSESSMENT"/ OR "SYMPTOM ASSESSMENT"/ OR "DIAGNOSTIC SELF EVALUATION"/ OR "INVESTIGATIVE TECHNIQUES"/ OR "TECHNOLOGY ASSESSMENT, BIOMEDICAL"/ OR "MEDICAL INFORMATICS"/ OR "POINT-OF-CARE SYSTEMS"/ OR "MACHINE LEARNING"/ OR "MOBILE APPLICATIONS"/ OR "CELL PHONE"/ OR SMARTPHONE/ OR "COMPUTERS, HANDHELD"/ OR "SOFTWARE VALIDATION"/ OR "MONITORING, PHYSIOLOGIC"/ OR ACCELEROMETRY/ OR ACTIGRAPHY/ OR "WEARABLE ELECTRONIC DEVICES"/ OR "REMOTE SENSING TECHNOLOGY"/ OR TELEMETRY/ OR "VIRTUAL REALITY"/ OR SOFTWARE/ OR THERMOGRAPHY/) OR sensor OR sensors OR nonwearab* OR "non-wearab*" OR webcam* OR "structured light" OR "artificial intelligence" OR assessment OR evaluation OR "cloud computing" OR telerehabilitat* OR "tele rehabilitat*" OR "machine learning" OR kinesis OR smartphone* OR "smart phone*" OR (mobile* ADJ3 (technolog* OR device* OR phone*)) OR cellphone* OR "cell phone*" OR "speech recognition" OR software OR platform* OR accelerometer* OR gyroscope* OR magnetometer* OR actigraph* OR wearab* OR device* OR "big data" OR "internet of things" OR "closed loop" OR hybrid OR (home* ADJ5 monitor*) OR quantitative OR algorithm* OR

telemetr* OR instrumented OR "virtual reality" OR nonmobile OR portab* OR nonportab* OR technolog* OR (static ADJ3 (technolog* OR device*)).mp

AND

(exp "PARKINSONIAN DISORDERS"/ OR "BASAL GANGLIA DISEASES"/ OR (parkinson*).mp

AND

(wearab* OR nonwearab* OR home OR homes OR homelike OR homebased OR naturalistic OR ecologic* OR “free living”).mp

AND

limit to yr="2000 -Current"
