# Supplementary material for: Systematic Review Looking at the Use of Technology to Measure Free-Living Symptom and Activity Outcomes in Parkinson’s Disease in the Home or a Home-like Environment
Source: J Parkinsons Dis. 2020 Apr 3;10(2):429–54. doi: 10.3233/JPD-191781 (PMC7242826; doi:10.3233/JPD-191781)
Supplement: Supplementary Table 2 [file jpd-10-jpd191781-s002.pdf]

Supplementary Table 2. Table listing excluded papers and reasons for exclusion

Title	Authors	Published Year	Journal	Volume	Issue	Pages	Accession Number	DOI	Study	Notes
App-Based Bradykinesia Tasks for Clinic and Home Assessment in Parkinson's Disease: Reliability and Responsiveness	Heldman, D. A.; Urrea-Mendoza, E.; Lovera, L. C.; Schmerler, D. A.; Garcia, X.; Mohammad, M. E.; McFarlane, M. C. U.; Giuffrida, J. P.; Espay, A. J.; Fernandez, H. H.	2017	Journal of Parkinson's Disease	7	4	741-747	WOS:000416092thou20	10.3233/jpd-171159	Heldman 2017	Exclusion reason: Structured assessments (as opposed to free-living); Catherine Morgan (2018-06-13 20:49:26)(Screen): Again, a structured task as opposed to free-living;
3-day activity monitoring confirms inter-ictal alterations in patients with freezing of gait (FOG)	Hausdorff, J. M.; Weiss, A.; Herman, T.; Marina, B.; Ariel, T.; Nir, G.	2014	Movement Disorders	29	Suppl 1	S379		http://dx.doi.org/10.1002/mds.25914	Hausdorff 2014	Exclusion reason: No full text available (abstract/poster only);
Quantifying daily living transitions in patients with Parkinson's disease using a body-fixed sensor	Hausdorff, J. M.; Bernad-Elazari, H.; Herman, T.; Mirelman, A.; Gazit, E.; Giladi, N.	2016	Movement Disorders	31	Suppl 2	S247		http://dx.doi.org/10.1002/mds.26688	Hausdorff 2016	Exclusion reason: No full text available (abstract/poster only);
Ambulatory activity monitoring: Capturing participation-level decline in Parkinson's disease	Dibble, L. E.; Cavanaugh, J. T.; Earhart, G.; Ellis, T.; Ford, M. P.; Foreman, K. B.	2012	Movement Disorders	27	Suppl 1	S114		http://dx.doi.org/10.1002/mds.25051	Dibble 2012	Exclusion reason: No full text available (abstract/poster only); Catherine Morgan (2018-07-11 18:43:08)(Select): poster only; Catherine Morgan (2018-07-11 18:41:53)(Select): No full text ?poster;
Smart home technology for safety and functional independence: The UK experience	Dewsbury, G.; Linskell, J.	2011	NeuroRehabilitation	28	3	249-260		http://dx.doi.org/10.3233/NRE-2011-0653	Dewsbury 2011	Exclusion reason: No full text available (abstract/poster only); Catherine Morgan (2018-06-13 17:27:34)(Screen): no specific technology investigated, commentary (but sounds interesting);
Free-living monitoring of Parkinson's disease: Lessons from the field	Del Din, S.; Godfrey, A.; Mazza, C.; Lord, S.; Rochester, L.	2016	Movement Disorders	31	9	1293-1313	WOS:000383773600048	10.1002/mds.26718	Del Din 2016	Exclusion reason: No full text available (abstract/poster only);
Comparison of effect of variable deep brain stimulation (DBS) frequencies on gait and balance in Parkinson's disease (PD) patients with either bilateral GPi or STN stimulators employing wearable wireless sensors	Deep, A.; Lieberman, A.; Dhall, R.; Lockhart, T.; Frames, C.; Shafer, S.; Tateuchi, N.; Simpson, E.; McCauley, M.; Krishnamurthi, N.	2016	Movement Disorders	31	Suppl 2	S23-S24		http://dx.doi.org/10.1002/mds.26688	Deep 2016	Exclusion reason: No full text available (abstract/poster only);

Automatic assessment of the motor state of Parkinson's disease patients	Czyzewski, A.; Kostek, B.; Slawek, J.; Kaszuba, K.	2012	Parkinsonism and Related Disorders	18	SUPPL. 2	S101-S102	http://dx.doi.org/10.1016/S1353-8020%2811%2970475-8	Czyzewski 2012	Exclusion reason: No full text available (abstract/poster only); Catherine Morgan (2018-07-10 00:15:13)(Select): Abstract only;	
Prescribed gait tests versus continuous monitoring of gait in people with Parkinson's disease	Curtze, C.; McNames, J.; El-Gohary, M.; Nutt, J. G.; Mancini, M.; Carlson-Kuhta, P.; Horak, F. B.	2016	Movement Disorders	31	Suppl 2	S181-S182	http://dx.doi.org/10.1002/mds.26688	Curtze 2016	Exclusion reason: No full text available (abstract/poster only); Catherine Morgan (2018-07-10 00:13:03)(Select): poster;	
Naturalistic Assessment of Everyday Functioning in Individuals with Parkinson's disease	Cunningham, R.; Schmitter-Edgecombe, M.	2017	Archives of Clinical Neuropsychology	32	6		WOS:000414346100206	10.1093/arclin/acx076.32	Cunningham 2017	Exclusion reason: No full text available (abstract/poster only); Catherine Morgan (2018-07-10 00:11:58)(Select): No full text that I could find, the article itself indeed was not found on journal search or google scholar...; Catherine Morgan (2018-07-10 00:11:58)(Select): No full text that I could find, the article itself indeed was not found on journal search or google scholar...; Catherine Morgan (2018-06-11 23:22:33)(Screen): No abstract, so unclear what (if any) tech used;
Sensors, wearables and devices in the E-age: Tomorrow's world today	Cummins, P.; Serruys, P. W.	2015	EuroIntervention	10	12	1373-1374		http://dx.doi.org/10.4244/EIJV10,12A238	Cummins 2015	Exclusion reason: No full text available (abstract/poster only); Catherine Morgan (2018-07-09 23:57:29)(Select): No full text available, but this is a review I think;
Prospective study on cost-effectiveness of home-based motor assessment in Parkinson's disease	Cubo, E.; Mariscal, N.; Solano, B.; Becerra, V.; Armesto, D.; Calvo, S.; Arribas, J.; Seco, J.; Martinez, A.; Zorrilla, L.; Heldman, D.	2017	Journal of Telemedicine and Telecare	23	2	328-338	WOS:000394260400016	10.1177/1357633x16638971	Cubo 2017	Exclusion reason: Wrong outcomes: e.g., EEG, imaging only, molecular diagnostic/therapeutic techniques, EMG;
Associations between clinical and performance measures with self-restricted driving practices in older adults with Parkinson's disease	Crizzle, A. M.; Myers, A. M.; Almeida, Q. J.; Roy, E.	2013	Movement Disorders	28	Suppl 1	S380-S381		http://dx.doi.org/10.1002/mds.25605	Crizzle 2013	Exclusion reason: Vehicle driving outcome measures;
Video assessment of finger tapping for Parkinson's disease and other movement disorders	Criss, K.; McNames, J.	2011	Conference proceedings: Annual International Conference of the IEEE Engineering in	2011		7123-7126			Criss 2011	Exclusion reason: Structured assessments (as opposed to free-living); Catherine Morgan (2018-06-11 22:40:37)(Screen): Not tested in home-like environment yet?;

			Medicine and Biology Society							
Parkinson's disease: Streamlined clinical trials, from a home computer	Couzin, J.	2008	Science	320	5880	1143		http://dx.doi.org/10.1126/science.320.5880.1143	Couzin 2008	Exclusion reason: Wrong study design; Catherine Morgan (2018-06-11 23:13:22)(Screen): Unclear what this is about, no abstract;
A lightweight web of things open platform to facilitate context data management and personalized healthcare services creation	Corredor, I.; Metola, E.; Bernardos, A. M.; Tarrio, P.; Casar, J. R.	2014	International Journal of Environmental Research and Public Health	11	5	4676-4713	http://dx.doi.org/10.3390/ijerph110504676		Corredor 2014	Exclusion reason: Wrong study design; Catherine Morgan (2018-06-11 23:11:58)(Screen): Parkinson's disease? Need to check text;
Gait differences between initial symptom onset of tremor-dominant and non-tremor dominant sub-types in Parkinson's disease	Chomiak, T.; Pereira, F.; Luan, K.; Cihal, A.; Meyer, N.; Hu, B.	2016	Neurology	86	16 Suppl 1				Chomiak 2016	Exclusion reason: No full text available (abstract/poster only); Catherine Morgan (2018-07-09 23:15:23)(Select): Full text unavailable;
Toward Pervasive Gait Analysis With Wearable Sensors: A Systematic Review	Chen, S. S.; Lach, J.; Lo, B.; Yang, G. Z.	2016	IEEE Journal of Biomedical and Health Informatics	20	6	1521-1537	WOS:000389846700008	10.1109/jbhi.2016.2608720	Chen 2016	Exclusion reason: Wrong study design;
Detecting mobility for monitoring patients with Parkinson's disease at home using RSSI in a Wireless Sensor Network	Chakraborty, S.; Ghosh, S. K.; Jamthe, A.; Agrawal, D. P.	2013	4th International Conference on Ambient Systems, Networks and Technologies	19		956-961	WOS:000361480500120	10.1016/j.procs.2013.06.132	Chakraborty 2013	Exclusion reason: Wrong study design;
Portable objective assessment of upper extremity motor function in Parkinson's disease	Chahine, L. M.; Uribe, L.; Hogarth, P.; McNames, J.; Siderowf, A.; Marek, K.; Jennings, D.	2017	Parkinsonism & Related Disorders	43		61-66	WOS:000415393100010	10.1016/j.parkreldis.2017.07.017	Chahine 2017	Exclusion reason: Structured assessments (as opposed to free-living);
Home-Based Physical Behavior in Late Stage Parkinson Disease Dementia: Differences between Cognitive Subtypes	Cerff, B.; Maetzler, W.; Sulzer, P.; Kampmeyer, M.; Prinzen, J.; Hobert, M. A.; Blum, D.; van Lummel, R.; Del Din, S.; Graber, S.; Berg, D.; Liepelt-Scarfone, I.	2017	Neurodegenerative Diseases	17	04-May	135-144	WOS:000407170000003	10.1159/000460251	Cerff 2017	Exclusion reason: No full text available (abstract/poster only); Catherine Morgan (2018-07-09 22:40:32)(Select): Full text not available through UoB - looks like a good paper though - ask the authors for text or is this not fair on the other papers?;
A comprehensive motor symptom monitoring and management system: the bradykinesia case	Cancela, J.; Pansera, M.; Arredondo, M. T.; Estrada, J. J.; Pastorino, M.; Pastor-Sanz, L.; Villalar, J. L.	2010	Conference proceedings: Annual International Conference of the IEEE Engineering in	2010		1008-1011			Cancela 2010	Exclusion reason: Structured assessments (as opposed to free-living);

			Medicine and Biology Society							
Guidelines for the economic analysis of a telematic platform for Parkinson's disease monitoring	Cancela, J.; Arredondo, M. T.; Hurtado, O.; Ieee	2013	2013 IEEE 13th International Conference on Bioinformatics and Bioengineering				WOS:000335217700080			Exclusion reason: Wrong study design;
Deep learning for freezing of gait detection in Parkinson's disease patients in their homes using a waist-worn inertial measurement unit	Camps, J.; Sama, A.; Martin, M.; Rodriguez-Martin, D.; Perez-Lopez, C.; Arostegui, J. M. M.; Cabestany, J.; Catala, A.; Alcaine, S.; Mestre, B.; Prats, A.; Crespo-Maraver, M. C.; Counihan, T. J.; Browne, P.; Quinlan, L. R.; Laighin, G. O.; Sweeney, D.; Lewy, H.; Vainstein, G.; Costa, A.; Annicchiarico, R.; Bayes, A.; Rodriguez-Molinero, A.	2018	Knowledge-Based Systems	139		119-131	WOS:000417773400011	10.1016/j.knosys.2017.10.017	Camps 2018	Exclusion reason: Structured assessments (as opposed to free-living);
A device-based quantification and assessment of tremor in deep brain stimulation patients	Bremm, R. P.; Goncalves, J.; Koch, K. P.; Hertel, F.	2017	Stereotactic and Functional Neurosurgery	95	Suppl 1	194		http://dx.doi.org/10.1159/000478281	Bremm 2017	Exclusion reason: No full text available (abstract/poster only); Catherine Morgan (2018-07-04 23:07:04)(Select): Abstract only, also not clearly home setting and focussing on DBS input-output dynamics;
Can body worn cameras help us understand the complex relationship between physical activity and falls in people with Parkinson's?	Baker, K.; Harvey, M.; Monaghan, J.; Rochester, L.	2016	Movement Disorders	31	Suppl 2	S176		http://dx.doi.org/10.1002/mds.26688	Baker 2016	Exclusion reason: Wrong study design;
A wearable ultrasonic sensor network for analysis of bilateral gait symmetry	Ashhar, Karalikkadan; Cheong Boon, Soh; Keng He, Kong	2017	Conference proceedings: Annual International Conference of the IEEE Engineering in Medicine and Biology Society	2017		4455-4458	https://dx.doi.org/10.1109/EMBC.2017.8037845	Ashhar 2017		Exclusion reason: No full text available (abstract/poster only); Catherine Morgan (2018-07-03 00:34:51)(Select): Cannot get full-text access through UoB so can't check environment - may need to request full text; Catherine Morgan (2018-06-11 17:38:50)(Screen): Unclear whether this testing was in lab or at home?;
Detecting and monitoring the symptoms of Parkinson's disease using	Arora, S.; Venkataraman, V.; Zhan, A.; Donohue, S.; Biglan, K. M.;	2015	Parkinsonism & Related Disorders	21	6	650-653	WOS:000356105700018	10.1016/j.parkreldis.2015.02.026	Arora 2015	Exclusion reason: Structured assessments (as opposed to free-living);

smartphones: A pilot study	Dorsey, E. R.; Little, M. A.									
Parkinson disease assessment feasible with an at-home testing device	Anonymous	2009	Nature clinical practice. Neurology	5	3	124			Anonymous 2009	Exclusion reason: No full text available (abstract/poster only); Catherine Morgan (2018-07-02 23:41:33)(Select): Full-text review - can't seem to get hold of this;
Smart Gait-Aid Glasses for Parkinson's Disease Patients	Ahn, D.; Chung, H.; Lee, H. W.; Kang, K.; Ko, P. W.; Kim, N. S.; Park, T.	2017	IEEE Transactions on Biomedical Engineering	64	10	2394-2402	http://dx.doi.org/10.1109/TBME.2017.2655344	Ahn 2017		Exclusion reason: No full text available (abstract/poster only); Catherine Morgan (2018-07-02 20:58:25)(Select): ?environment;
Utilizing remote blood pressure monitoring in a phase III clinical drug trial for Parkinson's disease	Adams, J. L.; Biemiller, R. A.; Andrzejewski, K. L.; Sharma, S.; Rockhill, R. L.; Greco, B. L.; Hodgeman, K. C.; Singh, P.; Khanuja, S.; Simuni, T.; Biglan, K. M.	2016	Movement Disorders	31	Supplement 2	S687		http://dx.doi.org/10.1002/mds.26688	Adams 2016	Exclusion reason: No full text available (abstract/poster only);
Twenty Third Annual Symposium on Etiology, Pathogenesis, and Treatment of Parkinson's Disease and Other Movement Disorders	Anonymous	2009	Movement Disorders	24	12				Anonymous 2009	Exclusion reason: Wrong study design;
Review of first trial responses in balance control: influence of vestibular loss and Parkinson's disease	Allum, J. H. J.; Tang, K. S.; Carpenter, M. G.; Oude Nijhuis, L. B.; Bloem, B. R.	2011	Human movement science	30	2	279-95		https://dx.doi.org/10.1016/j.humov.2010.11.009	Allum 2011	Exclusion reason: No full text available (abstract/poster only); Catherine Morgan (2018-06-04 21:21:49)(Screen): Unclear whether FTRs measured by sensors, and in what environment, but could be interesting to look at;
Common daily activities in the virtual environment: a preliminary study in parkinsonian patients	Albani, G.; Pignatti, R.; Bertella, L.; Priano, L.; Semenza, C.; Molinari, E.; Riva, G.; Mauro, A.	2002	Neurological Sciences	23		S49-S50	WOS:000179317800001	10.1007/s100720200064	Albani 2002	Exclusion reason: No full text available (abstract/poster only); Catherine Morgan (2018-06-04 21:06:43)(Select): Odd, not sure what this technology is, small sample size;
A Survey on Ambient Intelligence in Healthcare	Acampora, G.; Cook, D. J.; Rashidi, P.; Vasilakos, A. V.	2013	Proceedings of the Ieee	101	12	2470-2494	WOS:000327606700005	10.1109/jproc.2013.2262913	Acampora 2013	Exclusion reason: Wrong study design; Catherine Morgan (2018-06-04 21:01:52)(Select): Not PD specifically but may contain a section on PD in full text?;
Wearable Wireless Inertial Sensors for Long-Time Monitoring of Specific Motor Symptoms in Parkinson's Disease	Lorenzi, P.; Rao, R.; Suppa, A.; Kita, A.; Parisi, R.; Romano, G.; Berardelli, A.; Irrera, F.	2015	BIODEVICES: Proceedings of the 2015 International Conference on Biomedical Electronics and Devices	168-173			WOS:000380546700026	10.5220/0005279201680173	Lorenzi 2015	Exclusion reason: Wrong setting;

A system for inference of spatial context of Parkinson's disease patients	Takac, B.; Catala, A.; Cabestany, J.; Chen, W.; Rauterberg, M.	2012	Studies in health technology and informatics	177		126-131			Takac 2012	Exclusion reason: Wrong study design;
Wearability assessment of a wearable system for Parkinson's disease remote monitoring based on a body area network of sensors	Cancela, J.; Pastorino, M.; Tzallas, A. T.; Tsipouras, M. G.; Rigas, G.; Arredondo, M. T.; Fotiadis, D. I.	2014	Sensors (Basel, Switzerland)	14	9	17235-17255		http://dx.doi.org/10.3390/s140917235	Cancela 2014	Exclusion reason: Wrong study design;
Quantitative home-based assessment of Parkinson's symptoms: The SENSE-PARK feasibility and usability study	Ferreira, J. J.; Godinho, C.; Santos, A. T.; Domingos, J.; Abreu, D.; Goncalves, N.; Barra, M.; Larsen, F.; Fagerbakke, O.; Akeren, I.; Wangen, H.; Serrano, A.; Weber, P.; Thorns, A.; Meckler, S.; Sollinger, S.; Van Uem, J.; Hobert, M. A.; Maier, K. S.; Matthew, H.; Isaacs, T.; Duffen, J.; Graessner, H.; Maetzler, W.	2015	Sinapse	15	1	180			Ferreira 2015	Exclusion reason: Wrong study design;
Body-Worn Sensors in Parkinson's Disease: Evaluating Their Acceptability to Patients	Fisher, J. M.; Hammerla, N. Y.; Rochester, L.; Andras, P.; Walker, R. W.	2016	Telemedicine and E-Health	22	1	63-69	WOS:000368448900010	10.1089/tmj.2015.0026	Fisher 2016	Exclusion reason: Wrong study design;
Mercury: A Wearable Sensor Network Platform for High-Fidelity Motion Analysis	Lorincz, K.; Chen, B. R.; Challen, G. W.; Chowdhury, A. R.; Patel, S.; Bonato, P.; Welsh, M.; Acm	2009	Sensys 09: Proceedings of the 7th Acm Conference on Embedded Networked Sensor Systems			183-196	WOS:000332845200014		Lorincz 2009	Exclusion reason: Wrong setting;
DyGo - Household Object Interaction Tracking for Smart Home Environments	Rosner, D.; Vasile, D.; Aungurencei, A.; Vasile, S.; Tanasiev, V.	2015	2015 20th International Conference on Control Systems and Computer Science	248-251			WOS:000380375200037	10.1109/cscs.2015.138	Rosner 2015	Exclusion reason: Wrong patient population;
Correlation of Quantitative Motor State Assessment Using a Kinetograph and Patient Diaries in Advanced PD: Data from an Observational Study	Ossig, C.; Gandor, F.; Fauser, M.; Bosredon, C.; Churilov, L.; Reichmann, H.; Horne, M. K.; Ebersbach, G.; Storch, A.	2016	Plos One	11	8		WOS:000382258100051	10.1371/journal.pone.0161559	Ossig 2016	Exclusion reason: Wrong setting;

Smart Meter Profiling For Health Applications	Chalmers, C.; Hurst, W.; Mackay, M.; Fergus, P.; Ieee	2015	2015 International Joint Conference on Neural Networks				WOS:000370730603134		Chalmers 2015	Exclusion reason: Wrong patient population;
Defining ambulatory bouts in free-living activity: Impact of brief stationary periods on bout metrics	Barry, G.; Galna, B.; Lord, S.; Rochester, L.; Godfrey, A.	2015	Gait & Posture	42	4	594-597	WOS:000366071500033	10.1016/j.gaitpost.2015.07.062	Barry 2015	Exclusion reason: Wrong patient population;
Quantitative demonstration of the efficacy of rotigotine transdermal patch in the treatment of nocturnal hypokinesia: A randomized, placebo-controlled trial using an axial inertial sensor	Bhidayasiri, R.; Sringean, J.; Chaiwong, S.; Anan, C.; Boonpang, K.; Sakdisornchai, K.; Jitkritsadakul, O.; Jagota, P.	2017	Movement Disorders	32	Suppl 2	975-976		http://dx.doi.org/10.1002/mds.27087	Bhidayasiri 2017	Exclusion reason: No full text available (abstract/poster only);
Feasibility of implementing a long-term mobile device program in PD patients	Bataille, L. R.; Herron, L. D.; Afek, M.; Feldman, I.; Admati, C.; Chowdhury, S.	2016	Movement Disorders	31	Suppl 2	S665		http://dx.doi.org/10.1002/mds.26688	Bataille 2016	Exclusion reason: No full text available (abstract/poster only);
Evaluating wearable sensors for objective measurement of motor features in Parkinson's disease and Huntington disease-a pilot study	Adams, J.; Xiong, M.; Dinesh, K.; Tarolli, C.; Sharma, S.; Sheth, N.; Aranyosi, A. J.; Zhu, W.; Goldenthal, S.; Biglan, K.; Dorsey, R.E.; Sharma, G.	2017	Movement Disorders	32	Suppl 2	444-445		http://dx.doi.org/10.1002/mds.27087	Adams 2017	Exclusion reason: No full text available (abstract/poster only);
A step forward to the future: UPDRS kinematic measures for telemedicine	Albani, G.; Azzaro, C.; Parisi, F.; Ferraris, C.; Giuberti, M.; Contin, L.; Pianu, D.; Pradotto, L.; Cimolin, V.; Cau, N.; Galli, M.; Nerino, R.; Ferrari, G.; Mauro, A.	2016	Movement Disorders	31	Suppl 2	S514		http://dx.doi.org/10.1002/mds.26688	Albani 2016	Exclusion reason: Wrong setting;
Development of a wireless sensor for tremors analysis	Alimonti, D.; Locatelli, P.; Caldara, M.; Re, V.	2016	Parkinsonism and Related Disorders	22	Suppl 2	e128-e129			Alimonti 2016	Exclusion reason: Structured assessments (as opposed to free-living); Catherine Morgan (2018-06-04 21:08:41)(Screen): Not sure about home-like environment setting;
Developing a new home monitoring device for dyskinesia in Parkinson's disease	Alty, J. E.; Cosgrove, J.; Lones, M. A.; Jamieson, S.; Duggan-Carter, P.; Peacey, C.; Wicks, C.; Naylor, R. F.; Turner, A. J.; Smith, S. L.	2016	Movement Disorders	31		S183-S183	WOS:000382559800547		Alty 2016	Exclusion reason: Wrong setting;

Home polysomnography in sleep related movement disorders	Anca-Herschkovitsch, M.	2010	Movement Disorders	25	Suppl 2	S406		http://dx.doi.org/10.1002/mds.23162	Anca-Herschkovitsch 2010	Exclusion reason: Wrong outcomes: e.g., EEG, imaging only, molecular diagnostic/therapeutic techniques, EMG; Catherine Morgan (2018-07-02 23:40:54)(Select): Full- text review - not sure if we should include PSG (EEG, EMG etc);
ESH 2016 Abstract	Anonymous	2016	Journal of Hypertension	34	Suppl 2				Anonymous 2016	Exclusion reason: Wrong outcomes: e.g., EEG, imaging only, molecular diagnostic/therapeutic techniques, EMG; Catherine Morgan (2018-06-04 21:32:34)(Screen): Amb BP monitoring mentioned - may be helpful;
Turnover movements using a wearable accelerometer in patients with different types of motor deficits	Anonymous	2016	Clinical Neurology	56	Suppl 1	S317			Anonymous 2016	Exclusion reason: Wrong setting;
Home monitoring in patients with advanced Parkinson's disease: A feasibility study. The CHRONIC consortium	Antonini, A.; Castiglioni, C.; Aguilo, J.; Farre, R.; Falco, A.; Pezzoli, G.	2002	Movement Disorders	17		S106-S107	WOS:000179489800364		Antonini 2002	Exclusion reason: No full text available (abstract/poster only); Catherine Morgan (2018-07-03 00:21:54)(Select): can't get hold of this - despite looking carefully through Movement Disorders 2002 issues...;
An external portable device for adaptive deep brain stimulation (aDBS) clinical research in advanced Parkinson's Disease	Arlotti, M.; Rossi, L.; Rosa, M.; Marceglia, S.; Priori, A.	2016	Medical Engineering & Physics	38	5	498-505	WOS:000375366400010	10.1016/j.medengphy.2016.02.007	Arlotti 2016	Exclusion reason: DBS developments or magnetic transcranial stimulation; Catherine Morgan (2018-06-04 21:42:39)(Screen): Unsure what the device measures but assume some parameter of clinical outcomes (?motor);
Detection of Motor Impairment in Parkinson's Disease Via Mobile Touchscreen Typing	Arroyo-Gallego, T.; Ledesma-Carbayo, M. J.; Sanchez-Ferro, A.; Butterworth, I.; Mendoza, C. S.; Matarazzo, M.; Montero, P.; Lopez-Blanco, R.; Puertas-Martin, V.; Trincado, R.; Giancardo, L.	2017	Ieee Transactions on Biomedical Engineering	64	9	1994-2002	WOS:000408111500002	10.1109/tbme.2017.2664802	Arroyo-Gallego 2017	Exclusion reason: Structured assessments (as opposed to free-living);
An Efficient Home-Based Risk of Falling Assessment Test Based on Smartphone and Instrumented Insole	Ayena, J. C.; Chapwouo, T. L. D.; Otis, M. J. D.; Menelas, B. A. J.; Ieee	2015	2015 Ieee International Symposium on Medical Measurements			416-421	WOS:000380610600078		Ayena 2015	Exclusion reason: Wrong setting;

			and Applications							
Home-Based Risk of Falling Assessment Test Using a Closed-Loop Balance Model	Ayena, J. C.; Zaibi, H.; Otis, M. J. D.; Menelas, B. A. J.	2016	Ieee Transactions on Neural Systems and Rehabilitation Engineering	24	12	1351-1362	WOS:000390559600009	10.1109/tnsre.2015.2508960	Ayena 2016	Exclusion reason: Wrong setting;
Late-breaking research a wearable accelerometer sensor system for unobtrusive real time monitoring of parkinson disease motor symptoms	Barnathan, M.; Khan, F.; Montgomery, M.; Myers, S.; Cote, L.; Loftus, S.	2012	Movement Disorders	27	4	e9		http://dx.doi.org/10.1002/mds.24987	Barnathan 2012	Exclusion reason: Wrong setting; Catherine Morgan (2018-07-03 00:53:12)(Select): Seems to be in lab, this was a poster at the 26th annual symposium on etiology, pathogenesis, and treatment of Parkinson disease and other movement disorders in Texas, 2012.; Catherine Morgan (2018-06-11 17:48:38)(Screen): At home or in lab?;
In-home quantitative mobility assessment pre and post medication adjustment in Parkinson's disease	Bee, C.; Jog, M.; Rahimi, F.; South, A.; Boissy, P.; Duval, C.	2011	Movement Disorders	26		S97-S98	WOS:000291359500294		Bee 2011	Exclusion reason: Structured assessments (as opposed to free-living); Catherine Morgan (2018-06-11 17:58:56)(Screen): No abstract...;
Using a wearable sensor to evaluate activity and motor response fluctuations in patients with Parkinson's disease (PD): Preliminary findings	Bernad-Elazari, H.; Weiss, A.; Oren, S.; Cohen, Y.; Mirelman, A.; Giladi, N.; Hausdorff, J.	2015	Movement Disorders	30	Suppl 1	S265		http://dx.doi.org/10.1002/mds.26295	Bernad-Elazari 2015	Exclusion reason: No full text available (abstract/poster only); Catherine Morgan (2018-07-04 22:00:47)(Select): Abstract only; Catherine Morgan (2018-07-04 19:33:54)(Select): Abstract only as preliminary findings;
Clinical Assessments in Parkinson's Disease: Scales and Monitoring	Bhidayasiri, R.; Martinez-Martin, P.	2017	International Review of Neurobiology	132		129-182		http://dx.doi.org/10.1016/bs.irn.2017.01.001	Bhidayasiri 2017	Exclusion reason: Wrong study design;
Impaired bed mobility: quantitative torque analysis with axial inertial sensors	Bhidayasiri, R.; Sringean, J.; Thanawattano, C.	2017	Neurodegenerative Disease Management	7	4	235-243	WOS:000410786000002	10.2217/nmt-2017-0016	Bhidayasiri 2017	Exclusion reason: No full text available (abstract/poster only); Catherine Morgan (2018-07-04 22:38:10)(Select): No full text via UoB or search engine (google scholar), or in UoB print catalogue;
Getting a good night sleep? The importance of recognizing and treating nocturnal hypokinesia in Parkinson's disease	Bhidayasiri, R.; Trenkwalder, C.	2018	Parkinsonism and Related Disorders	50		Oct-18		http://dx.doi.org/10.1016/j.parkreldis.2018.01.008	Bhidayasiri 2018	Exclusion reason: Wrong study design;
A mobile medical monitoring system: Concept, design and deployment	Biemer, M.; Hampe, J. F.	2005	ICMB 2005: International Conference on Mobile Business			464-471	WOS:000231259600069	10.1109/icmb.2005.9	Biemer 2005	Exclusion reason: Technology requiring clinician interaction;

Utilizing remote blood pressure monitoring in a phase III clinical drug trial for Parkinson's disease	Biemiller, R. A.; Andrzejewski, K. J.; Bull, M. T.; Helles, K.; Greco, B.; Oakes, D.; Simuni, T.; Biglan, K. M.	2015	Movement Disorders	30	Suppl 1	S71		http://dx.doi.org/10.1002/mds.26295	Biemiller 2015	Exclusion reason: Wrong outcomes: e.g., EEG, imaging only, molecular diagnostic/therapeutic techniques, EMG; Catherine Morgan (2018-07-04 22:54:38)(Select): Not new technology: using standard BP monitor which automatically transmits readings to clinicians/database;
Comparison of effect of variable deep brain stimulation (DBS) frequencies on gait and balance in parkinson disease (PD) patients with either bilateral GPI or STN stimulators employing wearable wireless sensors	Deep, A.; Liebermna, A.; Shafer, S.	2016	Annals of Neurology	80	Suppl 20	S104		http://dx.doi.org/10.1002/ana.24759	Deep 2016	Exclusion reason: Structured assessments (as opposed to free-living);
Accelerometer-Based Gait Assessment: Pragmatic Deployment On An International Scale	Del Din, S.; Hickey, A.; Woodman, S.; Hiden, H.; Morris, R.; Watson, P.; Nazarpour, K.; Catt, M.; Rochester, L.; Godfrey, A.; Ieee	2016	2016 IEEE Statistical Signal Processing Workshop				WOS:000390840200090		DelDin 2016	Exclusion reason: Wrong study design;
Automated assessment of advanced motor Parkinson's disease; a pilot study of the Parkinson's KinetiGraph as an objective tool for measurement of motor fluctuations	Bogdanova-Mihaylova, P.; Kavanagh, N.; Walsh, R. A.	2016	Movement Disorders	31	Suppl 2	S185		http://dx.doi.org/10.1002/mds.26688	Bogdanova-Mihaylova 2016	Exclusion reason: No full text available (abstract/poster only); Catherine Morgan (2018-07-04 22:59:43)(Select): Abstract for poster in MDS conference;
Quantitative assessment of parkinsonian tremor based on a linear acceleration extraction algorithm	Cai, G.; Lin, Z.; Dai, H.; Xia, X.; Xiong, Y.; Horng, S. J.; Lueth, T. C.	2018	Biomedical Signal Processing and Control	42		53-62		http://dx.doi.org/10.1016/j.bspc.2018.01.008	Cai 2018	Exclusion reason: Wrong setting; Catherine Morgan (2018-06-11 20:05:28)(Screen): home-based? doesn't look like it;
Technology and Parkinson's disease: State-of-the-art and trends in remote monitoring and rehabilitation	Cancela Gonzalez, J.; Pastorino, M.; Pastor-Sanz, L.; Gonzalez Marcos, A. P.; Arredondo Waldmeyer, M. T.	2012	Parkinsonism and Related Disorders	18	Suppl 2	S102		http://dx.doi.org/10.1016/S1353-8020%2811%2970476-X	CancelaGonzalez 2012	Exclusion reason: Wrong study design; Catherine Morgan (2018-07-09 20:35:39)(Select): Abstract only, review article so not appropriate anyway;
Testing feasibility and utility of remote data capture technology to assess Parkinson's disease	Carter, J.; Hellmers, N.; Hanineva, A.; Henchcliffe, C.	2016	Movement Disorders	31	Suppl 2	S187		http://dx.doi.org/10.1002/mds.26688	Carter 2016	Exclusion reason: Structured assessments (as opposed to free-living); Catherine Morgan (2018-07-09 21:00:53)(Select): abstract/poster only; Catherine

										Morgan (2018-06-11 22:20:38)(Screen): Not free-living, UPDRS essentially via technology;
A Web-Based System for Home Monitoring of Patients With Parkinson's Disease Using Wearable Sensors	Chen, B. R.; Patel, S.; Buckley, T.; Rednic, R.; McClure, D. J.; Shih, L.; Tarsy, D.; Welsh, M.; Bonato, P.	2011	IEEE Transactions on Biomedical Engineering	58	3	831-836	WOS:000287663100024	10.1109/tbme.2010.2090044	Chen 2011	Exclusion reason: Structured assessments (as opposed to free-living);
Analyzing Activity Behavior and Movement in a Naturalistic Environment Using Smart Home Techniques	Cook, D. J.; Schmitter-Edgecombe, M.; Dawadi, P.	2015	IEEE Journal of Biomedical and Health Informatics	19	6	1882-1892	WOS:000364857000013	10.1109/jbhi.2015.2461659	Cook 2015	Exclusion reason: Structured assessments (as opposed to free-living);
New smartphone system for personalized and at-home rehabilitation designed for people with Parkinson's disease	Gazit, E.; Dorfman, M.; Bezalel, P.; Ginis, P.; Nieuwboer, A.; Mazilu, S.; Ferrari, A.; Rocchi, L.; Mirelman, A.; Hausdorff, J. M.	2014	Movement Disorders	29		S150-S151	WOS:000337693401003		Gazit 2014	Exclusion reason: No full text available (abstract/poster only);
At-home testing battery for collecting objective motor data: Technology to capture longitudinal changes in Parkinson's disease	Goetz, C. G.; Stebbins, G. T.; Kubota, K.; Bronte-Stewart, H.; Elble, R. J.; Hallett, M.; Nutt, J.; Ramig, L.; Sanger, T.; DeLeeuw, W.; Dishman, E.; Wu, A.; Krauss, P.; Taylor, C. B.	2006	Movement Disorders	21		S121-S122	WOS:000235170100405		Goetz 2006	Exclusion reason: No full text available (abstract/poster only);
Parkinson's disease at-home testing device: Feasibility and relationship to UPDRS changes over six months	Goetz, C. G.; Stebbins, G. T.; Kubota, K.; DeLeeuw, W. C.; Bronte-Stewart, H.; Elble, R.; Hallett, M.; Nutt, J.; Romig, L.; Sanger, T.; Wu, A. D.; Kraus, P. H.; Blasucci, L. M.; Shamim, E. A.; Sethi, K.; Spielman, J.; Wolff, D.; Grove, A. S.; Taylor, C. B.	2008	Neurology	70	11	A57-A57	WOS:000257197200243		Goetz 2008	Exclusion reason: No full text available (abstract/poster only);
Evaluating Parkinson's disease patients at home: Utility of self-videotaping for objective motor, dyskinesia, and ON-OFF assessments	Goetz, C. G.; Leurgans, S.; Hinson, V. K.; Blasucci, L. M.; Zimmerman, J.; Fan, W. Q.; Nguyen, T.; Hsu, A.	2008	Movement Disorders	23	10	1479-1482	WOS:000258422300022	10.1002/mds.22127	Goetz 2008	Exclusion reason: Structured assessments (as opposed to free-living); Catherine Morgan (2018-06-13 20:05:14)(Screen): structured assessments (not free-living), but used at home;
Testing Objective Measures of Motor Impairment in Early Parkinson's Disease:	Goetz, C. G.; Stebbins, G. T.; Wolff, D.; DeLeeuw, W.; Bronte-Stewart, H.; Elble, R.; Hallet, M.; Nutt, J.;	2009	Movement Disorders	24	4	551-556	WOS:000265003800010	10.1002/mds.22379	Goetz 2009	Exclusion reason: Structured assessments (as opposed to free-living); Catherine Morgan (2018-06-13 20:07:21)(Screen): duplicated i think;

Feasibility Study of an At-Home Testing Device	Ramig, L.; Sanger, T.; Wu, A. D.; Kraus, P. H.; Blasucci, L. M.; Shamim, E. A.; Sethi, K. D.; Spielman, J.; Kubota, K.; Grove, A. S.; Dishman, E.; Taylor, C. B.									
The method of biofeedback training for the patients with Parkinson's disease	Goursky, I. S.; Likhachev, S. A.; Vashchylin, V. V.	2016	Movement Disorders	31	Suppl 2	S185		http://dx.doi.org/10.1002/mds.26688	Goursky 2016	Exclusion reason: Structured assessments (as opposed to free-living); Catherine Morgan (2018-06-13 19:07:59)(Screen): structured activity (not free-living), but carried out in person's own home (?) so interesting to us;
PERFORM: A system for the home monitoring and management of persons with neurodegenerative motor disorders	Greenlaw, R. B. L.; Konitsiotis, S.; Baga, D.; Bougia, P.; Pastor, M.; Pansera, M.; Fotiadis, D.	2010	Movement Disorders	25	Suppl 3	S649		http://dx.doi.org/10.1002/mds.23386	Greenlaw 2010	Exclusion reason: No full text available (abstract/poster only);
Unobtrusive Tremor Detection and Measurement via Human-Machine Interaction	Guttler, J.; Shah, R.; Georgoulas, C.; Bock, T.	2015	6th International Conference on Emerging Ubiquitous Systems and Pervasive Networks	63		467-474	WOS:000373842900061	10.1016/j.procs.2015.08.369	Guttler 2015	Exclusion reason: Wrong patient population;
Examination of naturalistic driving practices in drivers with Parkinson's disease compared to age and gender-matched controls	Crizzle, A. M.; Myers, A. M.	2013	Accident; analysis and prevention	50		724-731			Crizzle 2013	Exclusion reason: Vehicle driving outcome measures;
Associations Between Falls, Balance Confidence, Driving Speed, Braking, and Other Driving Practices in Parkinson ' s Disease	Crizzle, A. M.; Myers, A. M.; Roy, E. A.; Almeida, Q. J.	2015	Physical & Occupational Therapy in Geriatrics	33	1	72-86	WOS:000210516300006	10.3109/02703181.2014.991057	Crizzle 2015	Exclusion reason: Vehicle driving outcome measures;
Home-Based Monitoring and Assessment of Parkinson's Disease	Cunningham, L.; Mason, S.; Nugent, C.; Moore, G.; Finlay, D.; Craig, D.	2011	Ieee Transactions on Information Technology in Biomedicine	15	1	47-53	WOS:000286009500007	10.1109/titb.2010.2091142	Cunningham 2011	Exclusion reason: Structured assessments (as opposed to free-living); Catherine Morgan (2018-06-11 23:21:31)(Screen): This might be just a 'questionnaire/scale', in which case we can exclude it;
Computer-Based Assessment of Bradykinesia, Akinesia	Cunningham, L.; Nugent, C.; Moore, G.; Finlay, D.; Craig, D.	2009	Ambient Assistive Health and Wellness	5597		1-+	WOS:000270130800001		Cunningham 2009	Exclusion reason: Structured assessments (as opposed to free-living);

and Rigidity in Parkinson's Disease			Management in the Heart of the City, Proceeding							
Quantitative assessment of parkinsonian bradykinesia based on an inertial measurement unit	Dai, H.; Lin, H.; Lueth, T. C.	2015	BioMedical Engineering Online	14	1	68		http://dx.doi.org/10.1186/s12938-015-0067-8	Dai 2015	Exclusion reason: Structured assessments (as opposed to free-living); Catherine Morgan (2018-06-11 23:28:47)(Screen): Unclear if home-based?;
Quantitative Assessment of Parkinsonian Tremor Based on an Inertial Measurement Unit	Dai, H.; Zhang, P.; Lueth, T. C.	2015	Sensors (Basel, Switzerland)	15	10	25055-25071		http://dx.doi.org/10.3390/s151025055	Dai 2015	Exclusion reason: Structured assessments (as opposed to free-living); Catherine Morgan (2018-06-11 23:29:18)(Screen): ?duplicate ?home-based;
Exploring the use of wearable sensors to monitor drug response of patients with parkinson's disease in the home setting	Daneault, J. F.; Kanzler, C.; Lee, S.; Golabchi, F.; Vergara-Diaz, G.; Carvalho, G. F.; Fabara, E.; Sapienza, S.; Sudarsky, L.; Growdon, J.; Bonato, P.	2017	Neurology	88	16 Suppl 1				Daneault 2017	Exclusion reason: Structured assessments (as opposed to free-living);
Wrist sensor-based tremor severity quantification in Parkinson's disease using convolutional neural network	Kim, H. B.; Lee, W. W.; Kim, A.; Lee, H. J.; Park, H. Y.; Jeon, H. S.; Kim, S. K.; Jeon, B.; Park, K. S.	2018	Computers in Biology and Medicine	95		140-146		http://dx.doi.org/10.1016/j.compbiomed.2018.02.007	Kim 2018	Exclusion reason: Wrong setting; Catherine Morgan (2018-06-14 00:15:31)(Screen): Probably exclude as I think it's in a lab-based setting;
Ambulatory Activity Components Deteriorate Differently across Neurodegenerative Diseases: A Cross-Sectional Sensor-Based Study	Klenk, J.; Srulijes, K.; Schatton, C.; Schwickert, L.; Maetzler, W.; Becker, C.; Synofzik, M.	2016	Neurodegenerative Diseases	16	05-Jun	317-323	WOS:000383215200003	10.1159/000444802	Klenk 2016	Exclusion reason: No full text available (abstract/poster only);
Towards Mobile Gait Analysis: Concurrent Validity and Test-Retest Reliability of an Inertial Measurement System for the Assessment of Spatio-Temporal Gait Parameters	Kluge, Felix; Gasner, Heiko; Hannink, Julius; Pasluosta, Cristian; Klucken, Jochen; Eskofier, Bjorn M.	2017	Sensors (Basel, Switzerland)	17	7			https://dx.doi.org/10.3390/s17071522	Kluge 2017	Exclusion reason: Structured assessments (as opposed to free-living); Catherine Morgan (2018-06-14 00:31:37)(Screen): ?environment;
Community ambulation in people with Parkinson's disease: Relationships with walking performance, confidence and executive function	Lamont, R. M.; Morris, M. E.; Woollacott, M. H.; Brauer, S. G.	2012	Movement Disorders	27	Suppl 1	S298-S299		http://dx.doi.org/10.1002/mds.25051	Lamont 2012	Exclusion reason: No full text available (abstract/poster only);

Qualitative evaluation of the personal Kineti GraphTM movement recording system in a Parkinson's clinic	Langston, J. W.; Harsch, M.; Rees, L.; Dhall, R.; Gandhy, R.; Brillman, S.; Barlow, C.	2017	Movement Disorders	32	9	e9		http://dx.doi.org/10.1002/mds.27134	Langston 2017	Exclusion reason: Wrong outcomes: e.g., EEG, imaging only, molecular diagnostic/therapeutic techniques, EMG;
FIT: A Fog Computing Device for Speech Tele-Treatments	Monteiro, A.; Dubey, H.; Mahler, L.; Yang, Q.; Mankodiya, K.; Ieee	2016	2016 Ieee International Conference on Smart Computing			343-345	WOS:000390715200021		Monteiro 2016	Exclusion reason: Wrong setting;
Autonomous Quality Control of Joint Orientation Measured with Inertial Sensors	Lebel, K.; Boissy, P.; Nguyen, H.; Duval, C.	2016	Sensors	16	7		WOS:000380967000095	10.3390/s16071037	Lebel 2016	Exclusion reason: Wrong setting;
A 4 weeks home training program using a biofeedback serious game and sensors for Parkinson's disease	Leblong, E.; Fraudet, B.; Dandois, M.; Nicolas, B.; Gallien, P.; Ieee	2017	2017 International Conference on Virtual Rehabilitation				WOS:000411111900006		Leblong 2017	Exclusion reason: Structured assessments (as opposed to free-living);
A novel method for assessing the severity of levodopa-induced dyskinesia using wearable sensors	Lee, S. I.; Daneault, J. F.; Golabchi, F. N.; Patel, S.; Paganoni, S.; Shih, L.; Bonato, P.	2015	Conference proceedings: Annual International Conference of the IEEE Engineering in Medicine and Biology Society	2015		8087-8090		http://dx.doi.org/10.1109/EMBC.2015.7320270	Lee 2015	Exclusion reason: Structured assessments (as opposed to free-living);
Objective Measurement and Characterization of Sleep Benefit in Parkinson's Disease	Lee, W.; Evans, A. H.; Williams, D. R.	2017	Movement Disorders Clinical Practice	4	4	590-596	WOS:000407617600018	10.1002/mdc3.12489	Lee 2017	Exclusion reason: Structured assessments (as opposed to free-living);
Validation of a smartphone application measuring bradykinesia in Parkinson's disease	Lee, W.; Evans, A.; Williams, D. R.	2015	Movement Disorders	30	SUPPL. 1	S276		http://dx.doi.org/10.1002/mds.26295	Lee 2015	Exclusion reason: Structured assessments (as opposed to free-living);
Validation of a Smartphone Application Measuring Motor Function in Parkinson's Disease	Lee, W.; Evans, A.; Williams, D. R.	2016	Journal of Parkinson’s Disease	6	2	371-382	WOS:000378352200010	10.3233/jpd-150708	Lee 2016	Exclusion reason: Structured assessments (as opposed to free-living);
Quantification of Parkinson's disease characteristics using wireless accelerometers	LeMoyne, R.; Coroian, C.; Mastroianni, T.; Ieee	2009	2009 Icme International Conference on Complex Medical Engineering	480-+			WOS:000272211300095		LeMoyne 2009	Exclusion reason: Wrong setting;
Successful passive monitoring of early-stage Parkinson's disease	Lipsmeier, F.; Fernandez Garcia, I.; Wolf, D.; Kilchenmann, T.;	2017	Movement Disorders	32	Supplement 2	358-359		http://dx.doi.org/10.1002/mds.27087	Lipsmeier 2017	Exclusion reason: No full text available (abstract/poster only);

patient mobility in Phase I RG7935/PRX002 clinical trial with smartphone sensors	Scotland, A.; Schjodt-Eriksen, J.; Cheng, W. Y.; Siebourg-Polster, J.; Jin, L.; Soto, J.; Verselis, L.; Martin Facklam, M.; Boess, F.; Koller, M.; Grundman, M.; Little, M.; Monsch, A.; Postuma, R.; Gosh, A.; Kremer, T.; Taylor, K.; Czech, C.; Gossens, C.; Lindemann, M.									
Remote, high-frequency monitoring of motor symptoms in early-stage parkinson's disease patients in the phase I RG7935/PRX002 clinical trial	Lipsmeier, F.; Fernandez, G.; Wolf, D.; Kilchenmann, T.; Scotland, A.; Schjodt-Eriksen, J.; Cheng, W.; Siebourg-Polster, J.; Liping, J.; Soto, J.; Verselis, L.; Martin Facklam, M.; Boess, F.; Koller, M.; Grundman, M.; Little, M. A.; Monsch, A. U.; Postuma, R.; Ghosh, A.; Kremer, T.; Taylor, K. I.; Czech, C.; Gossens, C.; Lindemann, M.	2017	Neurodegenerative Diseases	17	Supplement 1	568			Lipsmeier 2017	Exclusion reason: No full text available (abstract/poster only);
A New Evolutionary Algorithm-Based Home Monitoring Device for Parkinson's Dyskinesia	Lones, M. A.; Alty, J. E.; Cosgrove, J.; Duggan-Carter, P.; Jamieson, S.; Naylor, R. F.; Turner, A. J.; Smith, S. L.	2017	Journal of Medical Systems	41	11		WOS:000413672500008	10.1007/s10916-017-0811-7	Lones 2017	Exclusion reason: Wrong setting;
Telemedicine and Parkinson's disease: At Patient's home therapeutic drug monitoring	Lopane, G.; Corzani, M.; Mellone, S.; Tacconi, C.; Chiari, L.; Cortelli, P.; Contin, M.	2017	Gait and Posture	57	Supplement 3	19		http://dx.doi.org/10.1016/j.gaitpost.2017.07.072	Lopane 2017	Exclusion reason: Structured assessments (as opposed to free-living);
Dyskinesia Detection and Monitoring by a Single Sensor in Patients With Parkinson's Disease	Lopane, G.; Mellone, S.; Chiari, L.; Cortelli, P.; Calandra-Buonaura, G.; Contin, M.	2015	Movement Disorders	30	9	1267-1271	WOS:000360759000016	10.1002/mds.26313	Lopane 2015	Exclusion reason: Structured assessments (as opposed to free-living);
Concurrent validity of accelerometry to measure gait in Parkinsons Disease	Lord, S.; Rochester, L.; Baker, K.; Nieuwboer, A.	2008	Gait & Posture	27	2	357-359	WOS:000253331300025	10.1016/j.gaitpost.2007.04.001	Lord 2008	Exclusion reason: Structured assessments (as opposed to free-living);
Executive dysfunction and attention contribute to gait interference in 'off' state Parkinson's Disease	Lord, S.; Rochester, L.; Hetherington, V.; Allcock, L. M.; Burn, D.	2010	Gait & Posture	31	2	169-174	WOS:000274584800005	10.1016/j.gaitpost.2009.09.019	Lord 2010	Exclusion reason: Structured assessments (as opposed to free-living);

Mobile Devices for the Real-Time Detection of Specific Human Motion Disorders	Lorenzi, P.; Rao, R.; Romano, G.; Kita, A.; Irrera, F.	2016	Ieee Sensors Journal	16	23	8220-8227	WOS:000388218100006	10.1109/jsen.2016.2530944	Lorenzi 2016	Exclusion reason: Wrong setting;
Smart sensing systems for the detection of human motion disorders	Lorenzi, P.; Rao, R.; Romano, G.; Kita, A.; Serpa, M.; Filesi, F.; Parisi, R.; Suppa, A.; Bologna, M.; Berardelli, A.; Irrera, F.	2015	Eurosensors 2015	120		324-327	WOS:000380499300073	10.1016/j.proeng.2015.08.626	Lorenzi 2015	Exclusion reason: Wrong setting;
New strides in wearable sensor technology	Mackinnon, C. D.	2013	Movement Disorders	28	8	1025-1026		http://dx.doi.org/10.1002/mds.25468	Mackinnon 2013	Exclusion reason: Wrong study design;
Quantitative analysis of anticipatory postural adjustment in patients with Parkinson's disease	Maier, K. S.; Nusbaum, S.; Elshehabi, M.; Hobert, M. A.; Hasmann, S. E.; Berg, D.; Maetzler, W.	2015	Clinical Neurophysiology	126	8	e162-e163		http://dx.doi.org/10.1016/j.clinph.2015.04.272	Maier 2015	Exclusion reason: No full text available (abstract/poster only); Catherine Morgan (2018-06-14 19:52:31)(Screen): environment? i suspect this is lab-based;
Continuous monitoring of turning mobility in Parkinson's disease	Mancini, M.; El-Gohary, M.; Nutt, J. G.; Horak, F. B.	2014	Movement Disorders	29	Suppl 1	S165-S166		http://dx.doi.org/10.1002/mds.25914	Mancini 2014	Exclusion reason: No full text available (abstract/poster only);
A Wearable Assistant for Gait Training for Parkinson's Disease with Freezing of Gait in Out-of-the-Lab Environments	Mazilu, S.; Blanke, U.; Dorfman, M.; Gazit, E.; Mirelman, A.; Hausdorff, J. M.; Troster, G.	2015	Acm Transactions on Interactive Intelligent Systems	5	1		WOS:000360088100005	10.1145/2701431	Mazilu 2015	Exclusion reason: Therapeutic approach of technologies (not outcome measurement);
Prediction of Freezing of Gait in Parkinson's From Physiological Wearables: An Exploratory Study	Mazilu, S.; Calatroni, A.; Gazit, E.; Mirelman, A.; Hausdorff, J. M.; Troster, G.	2015	IEEE journal of biomedical and health informatics	19	6	1843-1854		http://dx.doi.org/10.1109/JBHI.2015.2465134	Mazilu 2015	Exclusion reason: Structured assessments (as opposed to free-living);
Dopaminergic-induced dyskinesia assessment based on a single belt-worn accelerometer	Perez-Lopez, C.; Sama, A.; Rodriguez-Martin, D.; Moreno-Arostegui, J. M.; Cabestany, J.; Bayes, A.; Mestre, B.; Alcaine, S.; Quispe, P.; Laighin, G. O.; Sweeney, D.; Quinlan, L. R.; Counihan, T. J.; Browne, P.; Annicchiarico, R.; Costa, A.; Lewy, H.; Rodriguez-Molinero, A.	2016	Artificial Intelligence in Medicine	67		47-56	WOS:000374078900004	10.1016/j.artmed.2016.01.001	Perez-Lopez 2016	Exclusion reason: Structured assessments (as opposed to free-living);
Deep learning in objective classification of spontaneous movement of patients with Parkinson's disease using large-scale free-living sensor data	Pfister, F.; Kulic, D.; Um, T.; Pichler, D.; Ahmadi, A.; Lang, M.; Konig, G.; Achilles, F.; Endo, S.; Abedinpour, K.; Ziegler, K.; Botzel, K.; Hirche, S.; Ceballos-Baumann, A.; Fietzek, U.	2017	Movement Disorders	32	Suppl 2	912		http://dx.doi.org/10.1002/mds.27087	Pfister 2017	Exclusion reason: No full text available (abstract/poster only);

Validation of a step detection Algorithm during straight Walking and turning in Patients with Parkinson's disease and older Adults Using an Inertial Measurement Unit at the Lower Back	Pham, M. H.; Elshehabi, M.; Haertner, L.; Del Din, S.; Srulijes, K.; Heger, T.; Synofzik, M.; Hobert, M. A.; Faber, G. S.; Hansen, C.; Salkovic, D.; Ferreira, J. J.; Berg, D.; Sanchez-Ferro, A.; van Dieen, J. H.; Becker, C.; Rochester, L.; Schmidt, G.; Maetzler, W.	2017	Frontiers in Neurology	8			WOS:000409090800002	10.3389/fneur.2017.00457	Pham 2017	Exclusion reason: Structured assessments (as opposed to free-living);
Algorithm for Turning Detection and Analysis Validated under Home-Like Conditions in Patients with Parkinson's Disease and Older Adults using a 6 Degree-of-Freedom Inertial Measurement Unit at the Lower Back	Pham, M. H.; Elshehabi, M.; Haertner, L.; Heger, T.; Hobert, M. A.; Faber, G. S.; Salkovic, D.; Ferreira, J. J.; Berg, D.; Sanchez-Ferro, A.; van Dieen, J. H.; Maetzler, W.	2017	Frontiers in Neurology	8			WOS:000398829300001	10.3389/fneur.2017.00135	Pham 2017	Exclusion reason: Structured assessments (as opposed to free-living);
Acquisition, validation and preprocessing of wrist-worn sensor data in patients with Parkinson's disease and healthy controls	Pichler, D.; Lang, M.; Kulic, D.; Pfister, F.; Konig, G.; Um, T.; Ahmadi, A.; Endo, S.; Achilles, F.; Abedinpour, K.; Botzel, K.; Ceballos-Baumann, A.; Hirche, S.; Fietzek, U.	2017	Movement Disorders	32	Suppl 2	912-913		http://dx.doi.org/10.1002/mds.27087	Pichler 2017	Exclusion reason: No full text available (abstract/poster only);
Automatic recognition of gait-related health problems in the elderly using machine learning	Pogorelc, B.; Bosnic, Z.; Gams, M.	2012	Multimedia Tools and Applications	58	2	333-354	WOS:000302484500003	10.1007/s11042-011-0786-1	Pogorelc 2012	Exclusion reason: Wrong setting;
Continuous motion sensor assessment of parkinson's disease during activities of daily living	Pulliam, C.; Heldman, D.; Brokaw, E.; Burack, M.; Mera, T.	2015	Neurology	84	Suppl 14				Pulliam 2015	Exclusion reason: Structured assessments (as opposed to free-living); Catherine Morgan (2018-06-20 21:04:33)(Screen): Probably not - this is a laboratory environment it would seem, although they say 'home-simulated';
Continuous Assessment of Levodopa Response in Parkinson's Disease Using Wearable Motion Sensors	Pulliam, C. L.; Heldman, D. A.; Brokaw, E. B.; Mera, T. O.; Mari, Z. K.; Burack, M. A.	2018	IEEE Transactions on Biomedical Engineering	65	1	159-164	WOS:000418722000018	10.1109/tbme.2017.2697764	Pulliam 2018	Exclusion reason: Structured assessments (as opposed to free-living); Catherine Morgan (2018-06-20 21:05:27)(Screen): Again, this seems to be essentially a lab environment;
Characterization of gait freezing in Parkinson's disease using a novel	Rahimi, F.; South, A.; Bell-Boucher, D.; Bapat,	2012	Movement Disorders	27		S509-S509	WOS:000305507704326		Rahimi 2012	Exclusion reason: No full text available (abstract/poster only);

foot-sensor based methodology in laboratory and in patients' homes	P.; Mohammad, Y.; Zhu, L.; Vyas, M.; Jog, M.									
Pre-post treatment effect of rasagiline on freezing of gait during controlled and free walking	Rahimi, F.; South, A.; Bell-Boucher, D.; Bapat, P.; Mohammed, Y.; Vyas, M.; Jog, M.; Zhu, L.	2012	Movement Disorders	27	Suppl 1	S273		http://dx.doi.org/10.1002/mds.25051	Rahimi 2012	Exclusion reason: No full text available (abstract/poster only);
Using ecological whole body kinematics to evaluate effects of medication adjustment in Parkinson disease	Rahimi, F.; Bee, C.; Duval, C.; Boissy, P.; Edwards, R.; Jog, M.	2014	Journal of Parkinson's Disease	4	4	617-627		http://dx.doi.org/10.3233/JPD-140370	Rahimi 2014	Exclusion reason: Structured assessments (as opposed to free-living); Catherine Morgan (2018-06-20 21:11:12)(Screen): We need to decide whether to be strict on free-living assessment, or allow these scripted activities;
Tremor UPDRS Estimation in home environment	Rigas, G.; Gatsios, D.; Fotiadis, D. I.; Chondrogiorgi, M.; Tsironis, C.; Konitsiotis, S.; Gentile, G.; Marcante, A.; Antonini, A.	2016	2016 38th Annual International Conference of the IEEE Engineering in Medicine and Biology Society			3642-3645	WOS:000399823503244		Rigas 2016	Exclusion reason: Structured assessments (as opposed to free-living);
Assessment of tremor activity in the Parkinson's disease using a set of wearable sensors	Rigas, G.; Tzallas, A. T.; Tsipouras, M. G.; Bougia, P.; Tripoliti, E. E.; Baga, D.; Fotiadis, D. I.; Tsouli, S. G.; Konitsiotis, S.	2012	IEEE transactions on information technology in biomedicine: a publication of the IEEE Engineering in Medicine and Biology Society	16	3	478-487		http://dx.doi.org/10.1109/TITB.2011.2182616	Rigas 2012	Exclusion reason: Structured assessments (as opposed to free-living); Catherine Morgan (2018-06-20 21:23:39)(Screen): environment?;
Analysis of subtle movements related to neurodegenerative diseases using wearable inertial sensors: a study in healthy subjects	Rigoberto, M. M.; Otniel, P. R.; Juan-Carlos, A. V.; Daniel, L. E.; Arturo, M. M.	2013	Conference proceedings: Annual International Conference of the IEEE Engineering in Medicine and Biology Society	2013		6119-6122		http://dx.doi.org/10.1109/EMBC.2013.6610949	Rigoberto 2013	Exclusion reason: Wrong setting; Catherine Morgan (2018-06-20 21:24:15)(Screen): environment?;
Comparison of Features, Window Sizes and Classifiers in Detecting Freezing of Gait in Patients with Parkinson's Disease Through a Waist-Worn Accelerometer	Rodriguez-Martin, D.; Sama, A.; Perez-Lopez, C.; Catala, A.; Mestre, B.; Alcaine, S.; Bayes, A.	2016	Artificial Intelligence Research and Development	288		127-136	WOS:000390305800018	10.3233/978-1-61499-696-5-127	Rodriguez-Martin 2016	Exclusion reason: Structured assessments (as opposed to free-living);

A wearable inertial measurement unit for long-term monitoring in the dependency care area	Rodriguez-Martin, D.; Perez-Lopez, C.; Sama, A.; Cabestany, J.; Catala, A.	2013	Sensors (Basel, Switzerland)	13	10	14079-14104		http://dx.doi.org/10.3390/s131014079	Rodriguez-Martin 2013	Exclusion reason: Wrong setting;
A Waist-Worn Inertial Measurement Unit for Long-Term Monitoring of Parkinson's Disease Patients	Rodriguez-Martin, D.; Perez-Lopez, C.; Sama, A.; Catala, A.; Arostegui, J. M. M.; Cabestany, J.; Mestre, B.; Alcaine, S.; Prats, A.; Crespo, M. D.; Bayes, A.	2017	Sensors	17	4		WOS:000400822900168	10.3390/s17040827	Rodriguez-Martin 2017	Exclusion reason: Wrong setting;
Home detection of freezing of gait using Support Vector Machines through a single waist-worn triaxial accelerometer	Rodriguez-Martin, D.; Sama, A.; Perez-Lopez, C.; Catala, A.; Arostegui, J. M. M.; Cabestany, J.; Bayes, A.; Alcaine, S.; Mestre, B.; Prats, A.; Crespo, M. C.; Counihan, T. J.; Browne, P.; Quinlan, L. R.; Laighin, G. O.; Sweeney, D.; Lewy, H.; Azuri, J.; Vainstein, G.; Annicchiarico, R.; Costa, A.; Rodriguez-Molinero, A.	2017	PLoS One	12	2	e0171764		http://dx.doi.org/10.1371/journal.pone.0171764	Rodriguez-Martin 2017	Exclusion reason: Structured assessments (as opposed to free-living);
Analysis of Correlation between an Accelerometer-Based Algorithm for Detecting Parkinsonian Gait and UPDRS Subscales	Rodriguez-Molinero, A.; Sama, A.; Perez-Lopez, C.; Rodriguez-Martin, D.; Alcaine, S.; Mestre, B.; Quispe, P.; Giuliani, B.; Vainstein, G.; Browne, P.; Sweeney, D.; Arostegui, J. M. M.; Bayes, A.; Lewy, H.; Costa, A.; Annicchiarico, R.; Counihan, T.; Laighin, G. O.; Cabestany, J.	2017	Frontiers in Neurology	8			WOS:000409085800001	10.3389/fneur.2017.00431	Rodriguez-Molinero 2017	Exclusion reason: Structured assessments (as opposed to free-living);
Comparing speech function in persons with Parkinson's disease in clinical and home environments	Rountrey, C. E.; Borras, N. M.; Ludlow, C. L.	2015	Movement Disorders	30	Suppl 1	S180		http://dx.doi.org/10.1002/mds.26295	Rountrey 2015	Exclusion reason: No full text available (abstract/poster only);
High resolution tracking of motor complications in Parkinson's disease	Roy, S. H.; Nawab, H. S.; Gilmore, D. L.; Cole, B. T.; Ganesan, S.; Thomas, C. A.; Saint-Hilaire, M. H.; Ellias, S. A.; De Luca, C. J.	2010	Movement Disorders	25	Suppl 2	S225		http://dx.doi.org/10.1002/mds.23162	Roy 2010	Exclusion reason: No full text available (abstract/poster only);

Autonomous tracking of body bradykinesia during unconstrained activities in Parkinson's disease	Roy, S.; Shiwani, B.; Kline, J.; Saint-Hilaire, M.; Thomas, C.; Gennert, M.; De Luca, G.	2017	Movement Disorders	32	Suppl 2	448-449		http://dx.doi.org/10.1002/mds.27087	Roy 2017	Exclusion reason: No full text available (abstract/poster only);
Quantification of tremor and bradykinesia in Parkinson's disease using a novel ambulatory monitoring system	Salarian, A.; Russmann, H.; Wider, C.; Burkhard, P. R.; Vingerhoets, F. J. G.; Aminian, K.	2007	IEEE Transactions on Biomedical Engineering	54	2	313-322		http://dx.doi.org/10.1109/TBME.2006.886670	Salarian 2007	Exclusion reason: Wrong setting;
Determining the optimal features in freezing of gait detection through a single waist accelerometer in home environments	Sama, A.; Rodriguez-Martin, D.; Perez-Lopez, C.; Catala, A.; Alcaine, S.; Mestre, B.; Prats, A.; Crespo, M. C.; Bayes, A.	2018	Pattern Recognition Letters	105		135-143	WOS:000428363000016	10.1016/j.patrec.2017.05.009	Sama 2018	Exclusion reason: Structured assessments (as opposed to free-living);
Saliva swallowing frequency measured using 24-hours ambulatory impedanceph monitoring in patients with parkinson's disease and drooling	Saskia, S.; Wim, H.; Hanneke, K.	2016	Dysphagia	31	6	796		http://dx.doi.org/10.1007/s00455-016-9752-4	Saskia 2016	Exclusion reason: No full text available (abstract/poster only); Catherine Morgan (2019-08-12 06:29:20)(Excluded): Again I did try this again but no luck;
Wearable sensors objectively measure gait parameters in Parkinson's disease	Schlachetzki, J. C. M.; Barth, J.; Marxreiter, F.; Gossler, J.; Kohl, Z.; Reinfelder, S.; Gassner, H.; Aminian, K.; Eskofier, B. M.; Winkler, J.; Klucken, J.	2017	PLoS One	12	10	e0183989		http://dx.doi.org/10.1371/journal.pone.0183989	Schlachetzki 2017	Exclusion reason: Structured assessments (as opposed to free-living);
Wearable sensor-based therapy titration for Parkinson's disease	Schmidt, R.; Heldman, D.; Hadley, A.; Riley, D.	2017	Movement Disorders	32	Suppl 2	445		http://dx.doi.org/10.1002/mds.27087	Schmidt 2017	Exclusion reason: No full text available (abstract/poster only);
Wearable electronics treat disease on the move	Sealy, C.	2014	Nano Today	9	3	262-263		http://dx.doi.org/10.1016/j.nantod.2014.04.004	Sealy 2014	Exclusion reason: Wrong study design;
Identification of activities of daily living in tremorous patients using inertial sensors	Serrano, J. I.; Lambrecht, S.; del Castillo, M. D.; Romero, J. P.; Benito-Leon, J.; Rocon, E.	2017	Expert Systems with Applications	83		40-48	WOS:000403030000004	10.1016/j.eswa.2017.04.032	Serrano 2017	Exclusion reason: Structured assessments (as opposed to free-living);
A new computer method for assessing drawing impairment in Parkinson's disease	Westin, J.; Ghiamati, S.; Memedi, M.; Nyholm, D.; Johansson, A.; Dougherty, M.; Groth, T.	2010	Journal of Neuroscience Methods	190	1	143-148	WOS:000279888800019	10.1016/j.jneumeth.2010.04.027	Westin 2010	Exclusion reason: Structured assessments (as opposed to free-living);
A home environment test battery for status assessment in patients with advanced Parkinson's disease	Westin, J.; Dougherty, M.; Nyholm, D.; Groth, T.	2010	Computer Methods and Programs in Biomedicine	98	1	27-35	WOS:000276544800003	10.1016/j.cmpb.2009.08.001	Westin 2010	Exclusion reason: Structured assessments (as opposed to free-living);

Real-time gait cycle parameter recognition using a wearable accelerometry system	Yang, C. C.; Hsu, Y. L.; Shih, K. S.; Lu, J. M.	2011	Sensors	11	8	7314-7326		http://dx.doi.org/10.3390/s110807314	Yang 2011	Exclusion reason: Structured assessments (as opposed to free-living); Catherine Morgan (2018-06-21 00:58:00)(Screen): environment?;
A novel device for long-term monitoring freezing-of-gait in people with Parkinson disease: Case study	Yang, W. C.; Chen, H. B.; Ding, J. J.; Lin, K. H.	2012	Journal of Neuroscience and Neuroengineering	1	2	243-247		http://dx.doi.org/10.1166/jnsne.2012.1028	Yang 2012	Exclusion reason: No full text available (abstract/poster only);
Development of a System for Quantitative Evaluation of Motor Function Using Kinect v2 Sensor	Yoshida, H.; Honda, T.; Lee, J.; Yano, S.; Kakei, S.; Kondo, T.; Ieee	2016	2016 International Symposium on Micro-Nanomechatronics and Human Science				WOS:000405001200082		Yoshida 2016	Exclusion reason: Structured assessments (as opposed to free-living);
The patient's perspective: The effect of levodopa on Parkinson symptoms	Zach, H.; Dirkx, M.; Pasman, J. W.; Bloem, B. R.; Helmich, R. C.	2017	Parkinsonism and Related Disorders	35		48-54		http://dx.doi.org/10.1016/j.parkreldis.2016.11.015	Zach 2017	Exclusion reason: Wrong setting; Catherine Morgan (2018-06-21 01:02:07)(Screen): environment?;
Assessing mobility at home in people with early Parkinson's disease using an instrumented Timed Up and Go test	Zampieri, C.; Salarian, A.; Carlson-Kuhta, P.; Nutt, J. G.; Horak, F. B.	2011	Parkinsonism & Related Disorders	17	4	277-280	WOS:000291073300011	10.1016/j.parkreldis.2010.08.001	Zampieri 2011	Exclusion reason: Structured assessments (as opposed to free-living);
The use of accelerometry as a tool to measure disturbed nocturnal sleep in Parkinson's disease	McGregor, S.; Churchward, P.; Soja, K.; O'Driscoll, D.; Braybrook, M.; Khodakarami, H.; Evans, A.; Farzanehfar, P.; Hamilton, G.; Horne, M.	2018	NPJ Parkinsons Disease	4			WOS:000422928100001	10.1038/s41531-017-0038-9	McGregor 2018	Exclusion reason: Wrong setting;
A web-based system for visualizing upper limb motor performance of Parkinson's disease patients	Memedi, M.; Bergqvist, U.; Westin, J.; Nyholm, D.	2013	Movement Disorders	28	Suppl 1	S112-S113		http://dx.doi.org/10.1002/mds.25605	Memedi 2013	Exclusion reason: Structured assessments (as opposed to free-living);
Automatic spiral analysis for objective assessment of motor symptoms in Parkinson's disease	Memedi, M.; Johansson, A.; Bergquist, F.; Nyholm, D.	2015	Movement Disorders	30	Suppl 1	S418		http://dx.doi.org/10.1002/mds.26295	Memedi 2015	Exclusion reason: Structured assessments (as opposed to free-living);
Self-reported symptoms and motor tests via telemetry in a 36-month levodopa-carbidopa intestinal gel infusion trial	Memedi, M.; Nyholm, D.; Johansson, A.; Palhagen, S.; Willows, T.; Widner, H.; Linder, J.; Westin, J.	2013	Movement Disorders	28	Suppl 1	S168		http://dx.doi.org/10.1002/mds.25605	Memedi 2013	Exclusion reason: Structured assessments (as opposed to free-living);
Spiral drawing during self-rated dyskinesia is	Memedi, M.; Westin, J.	2011	Movement Disorders	26	Suppl 2	S194-S195		http://dx.doi.org/10.1002/mds.23764	Memedi 2011	Exclusion reason: Structured assessments (as opposed to free-living);

more impared than during self-rated off										
Spatial and temporal variability during spirography	Memedi, M.; Westin, J.	2015	Movement Disorders	30	Suppl 1	S418		http://dx.doi.org/10.1002/mds.26295	Memedi 2015	Exclusion reason: Structured assessments (as opposed to free-living);
Automatic and objective assessment of alternating tapping performance in Parkinson's disease	Memedi, M.; Khan, T.; Grenholm, P.; Nyholm, D.; Westin, J.	2013	Sensors (Basel, Switzerland)	13	12	16965-16984			Memedi 2013	Exclusion reason: Structured assessments (as opposed to free-living);
Validity and Responsiveness of At-Home Touch Screen Assessments in Advanced Parkinson's Disease	Memedi, M.; Nyholm, D.; Johansson, A.; Palhagen, S.; Willows, T.; Widner, H.; Linder, J.; Westin, J.	2015	Ieee Journal of Biomedical and Health Informatics	19	6	1829-1834	WOS:000364857000007	10.1109/jbhi.2015.2468088	Memedi 2015	Exclusion reason: Structured assessments (as opposed to free-living);
Automatic Spiral Analysis for Objective Assessment of Motor Symptoms in Parkinson's Disease	Memedi, M.; Sadikov, A.; Groznik, V.; Zabkar, J.; Mozina, M.; Bergquist, F.; Johansson, A.; Haubenberger, D.; Nyholm, D.	2015	Sensors	15	9	23727-23744	WOS:000362512200139	10.3390/s150923727	Memedi 2015	Exclusion reason: Structured assessments (as opposed to free-living);
Comparison of actigraphy with polysomnographic studies in patients with Parkinson's disease	Memon, R.; Memon, A.; Joop, A.; Pilkington, J.; Wood, K.; Amara, A.	2017	Movement Disorders	32	Suppl 1			http://dx.doi.org/10.1002/mds.26972	Memon 2017	Exclusion reason: No full text available (abstract/poster only);
Feasibility of home-based automated Parkinson's disease motor assessment	Mera, T. O.; Heldman, D. A.; Espay, A. J.; Payne, M.; Giuffrida, J. P.	2012	Journal of Neuroscience Methods	203	1	152-156	WOS:000297896100019	10.1016/j.jneumeth.2011.09.019	Mera 2012	Exclusion reason: Structured assessments (as opposed to free-living);
Activity recognition with wearable sensors on loose clothing	Michael, B.; Howard, M.	2017	Plos One	12	10		WOS:000412163100008	10.1371/journal.pone.0184642	Michael 2017	Exclusion reason: Wrong setting;
Quantifying Timed-Up-and-Go Test: A Smartphone Implementation	Milosevic, M.; Jovanov, E.; Milenkovic, A.; Ieee	2013	2013 Ieee International Conference on Body Sensor Networks				WOS:000333337600022		Milosevic 2013	Exclusion reason: Structured assessments (as opposed to free-living);
Validation of actigraphy-derived sleep metrics in Parkinson's disease (PD) patients and elderly controls, taking into account clinical and physiological status	Munro, J.; Wolz, R.; Hill, D.; Dauvilliers, Y.	2017	Movement Disorders	32	Suppl 2	447-448		http://dx.doi.org/10.1002/mds.27087	Munro 2017	Exclusion reason: No full text available (abstract/poster only);
A Q-backpropagated time delay neural network for diagnosing severity of gait disturbances in Parkinson's disease	Nancy Jane, Y.; Khanna Nehemiah, H.; Arputharaj, K.	2016	Journal of Biomedical Informatics	60		169-176		http://dx.doi.org/10.1016/j.jbi.2016.01.014	NancyJane 2016	Exclusion reason: Wrong setting;
Design System to Remotely Monitor	Nguyen, H. T.; Vu, C. C.; Phan, V. Q.; Nguyen, V. D.; Nguyen, T. D.	2015	5th International Conference on	46		104-107	WOS:000357778100026	10.1007/978-3-319-11776-8_26	Nguyen 2015	Exclusion reason: Wrong setting;

Patients with Parkinson's Disease			Biomedical Engineering in Vietnam							
Using Inertial Sensors to Automatically Detect and Segment Activities of Daily Living in People With Parkinson's Disease	Nguyen, H.; Lebel, K.; Bogard, S.; Goubault, E.; Boissy, P.; Duval, C.	2018	Ieee Transactions on Neural Systems and Rehabilitation Engineering	26	1	197-204	WOS:000422939000022	10.1109/tnsre.2017.2745418	Nguyen 2018	Exclusion reason: Structured assessments (as opposed to free-living);
Home and health in people ageing with Parkinson's disease: study protocol for a prospective longitudinal cohort survey study	Nilsson, M. H.; Iwarsson, S.	2013	Bmc Neurology	13			WOS:000326193600001	10.1186/1471-2377-13-142	Nilsson 2013	Exclusion reason: Wrong outcomes: e.g., EEG, imaging only, molecular diagnostic/therapeutic techniques, EMG;
Wireless sensors to capture home and community walking for clinicaltrials and daily care	Norberg, A.; Dorsch, A.; Thomas, S.; Dobkin, B.	2013	Journal of Investigative Medicine	61	1	146-147		http://dx.doi.org/10.231/JIM.0b013e31827d3ac9	Norberg 2013	Exclusion reason: No full text available (abstract/poster only); Catherine Morgan (2019-08-12 06:25:19)(Excluded): Tried hard to get hold of but couldn't;
Wireless real-time electronic data capture for self-assessment of motor function and quality of life in Parkinson's disease	Nyholm, D.; Kowalski, J.; Aquilonius, S. M.	2004	Movement Disorders	19	4	446-451	WOS:000220849000011	10.1002/mds.10690	Nyholm 2004	Exclusion reason: Wrong outcomes: e.g., EEG, imaging only, molecular diagnostic/therapeutic techniques, EMG;
Longitudinal assessment of sleep in an incident Parkinson's disease cohort	O'Dowd, S. T.; Duncan, G. W.; Khoo, T. K.; Yarnall, A. J.; Lawson, R. A.; Burn, D. J.; Anderson, K. N.	2016	Movement Disorders	31	Suppl 2	S117		http://dx.doi.org/10.1002/mds.26688	O'Dowd 2016	Exclusion reason: No full text available (abstract/poster only);
Is Apple Watch a new hope for patients with Parkinson's disease?	Ohtsubo, H.; Iwaki, H.; Andou, R.; Tsujii, T.; Babe, H.; Nishikawa, N.; Nagai, M.; Nomoto, M.	2016	Movement Disorders	31	Suppl 2	S494-S495		http://dx.doi.org/10.1002/mds.26688	Ohtsubo 2016	Exclusion reason: No full text available (abstract/poster only);
In-home posture evaluation and visual feedback training to improve posture with a kinect-based system in Parkinson's disease: A case study	Okada, Y.; Shibata, T.; Tamei, T.; Orito, Y.; Funaya, H.; Obayashi, C.; Ikeda, K.; Hiyamizu, M.; Morioka, S.	2014	Movement Disorders	29		S256-S256	WOS:000337693401295		Okada 2014	Exclusion reason: No full text available (abstract/poster only);
Detection of falls in fluctuated Parkinson's disease: Combination of fall reports and motion recordings	Okuma, Y.; Mitoma, H.; Bloem, B. R.	2010	Movement Disorders	25	Suppl 2	S360		http://dx.doi.org/10.1002/mds.23162	Okuma 2010	Exclusion reason: No full text available (abstract/poster only);
Detection and quantification of freezing of gait and falls in	Okuma, Y.; Mitoma, H.; Yoneyama, M.	2015	Movement Disorders	30	Suppl 1	S44		http://dx.doi.org/10.1002/mds.26295	Okuma 2015	Exclusion reason: No full text available (abstract/poster only);

Parkinson's disease patients using a wearable motion sensor										
Identifying freezing of gait and falls in Parkinson's disease patients using a body-worn sensor	Okuma, Y.; Mitoma, H.; Yoneyama, M.	2016	Movement Disorders	31	Suppl 2	S246		http://dx.doi.org/10.1002/mds.26688	Okuma 2016	Exclusion reason: No full text available (abstract/poster only);
Identifying freezing of gait and falls in Parkinson's disease patients using a bodyworn sensor	Okuma, Y.; Mitoma, H.; Yoneyama, M.	2016	Clinical Neurology	56	Suppl 1	S278			Okuma 2016	Exclusion reason: No full text available (abstract/poster only);
Objective identification of freezing of gait in Parkinson's disease patients using a triaxial accelerometer	Okuma, Y.; Mitoma, H.; Yoneyama, M.	2017	Movement Disorders	32	Suppl 2	366		http://dx.doi.org/10.1002/mds.27087	Okuma 2017	Exclusion reason: No full text available (abstract/poster only);
A large observational study using mobile phone sensors reveals a personalized drug response in patients with Parkinson's Disease	Omberg, L.; Neto, E. C.; Perumal, T.; Bot, B. M.; Pratap, A.; Mangravite, L.	2017	Neurology	88	16 Suppl 1				Omberg 2017	Exclusion reason: Structured assessments (as opposed to free-living);
Empirical Wavelet Transform Based Features for Classification of Parkinson's Disease Severity	Oung, Q. W.; Muthusamy, H.; Basah, S. N.; Lee, H.; Vijean, V.	2018	Journal of Medical Systems	42	2	29		http://dx.doi.org/10.1007/s10916-017-0877-2	Oung 2018	Exclusion reason: Wrong setting;
Wearables in epilepsy and Parkinson's disease-A focus group study	Ozanne, A.; Johansson, D.; Hallgren Graneheim, U.; Malmgren, K.; Bergquist, F.; Alt Murphy, M.	2018	Acta Neurologica Scandinavica	137	2	188-194		http://dx.doi.org/10.1111/ane.12798	Ozanne 2018	Exclusion reason: Wrong outcomes: e.g., EEG, imaging only, molecular diagnostic/therapeutic techniques, EMG;
Sleep attacks in Parkinson's disease: a clinical and polysomnographic study	Pacchetti, C.; Martignoni, E.; Terzaghi, M.; Zangaglia, R.; Mancini, F.; Nappi, G.; Manni, R.	2003	Neurological Sciences	24	3	195-196	WOS:000186052700036	10.1007/s10072-003-0127-x	Pacchetti 2003	Exclusion reason: Wrong outcomes: e.g., EEG, imaging only, molecular diagnostic/therapeutic techniques, EMG;
A kinect-based physiotherapy and assessment platform for Parkinson's disease patients	Pachoulakis, I.; Xilourgos, N.; Papadopoulos, N.; Analyti, A.	2016	Journal of Medical Engineering	2016		9413642		http://dx.doi.org/10.1155/2016/9413642	Pachoulakis 2016	Exclusion reason: Structured assessments (as opposed to free-living);
Open-Access Electronic Diary for Motor Fluctuation and Dyskinesia Evaluation in Parkinson Disease:	Terroba-Chambi, C.; Bruno, V.; Medina-Escobar, A.; Nanni, F.; Cerquetti, D.; Rossi, M.; Merello, M.	2018	Clinical Neuropharmacology	41	1	20-22	WOS:000423714400005	10.1097/wnf.0000000000000264	Terroba-Chambi 2018	Exclusion reason: Wrong outcomes: e.g., EEG, imaging only, molecular diagnostic/therapeutic techniques, EMG;

Comparison With Paper Diary										
Can implementation of technology transform the management of Parkinson's? Lessons learnt from the Parkinson's KinetiGraphTM (PKGTM) service evaluation project	Thomas, C.; Mohammed, B.; Abdelgadir, E.; Silverdale, M. A.; Kobylecki, C.; Osborne, L.; Smith, M.; Hulejczuk, A.; Saha, A. R.; Bain, P. G.; Carroll, C.	2017	Movement Disorders	32	9	e10		http://dx.doi.org/10.1002/mds.27134	Thomas 2017	Exclusion reason: Therapeutic approach of technologies (not outcome measurement);
Automated dosing schemes for administration of microtablets of levodopa for Parkinson's disease, using wearable sensors	Thomas, I.; Bergquist, F.; Johansson, D.; Nyholm, D.; Memedi, M.; Westin, J.	2017	Movement Disorders	32	Suppl 2	914-915		http://dx.doi.org/10.1002/mds.27087	Thomas 2017	Exclusion reason: Structured assessments (as opposed to free-living);
Motor Performance Assessment in Parkinson's Disease: Association between Objective In-Clinic, Objective In-Home, and Subjective/Semi-Objective Measures	Toosizadeh, N.; Mohler, J.; Lei, H.; Parvaneh, S.; Sherman, S.; Najafi, B.	2015	PloS One	10	4		WOS:000353376800103	10.1371/journal.pone.0124763	Toosizadeh 2015	Exclusion reason: Structured assessments (as opposed to free-living); Catherine Morgan (2018-06-21 00:15:52)(Screen): Not sure if any tech used to measure PD outcomes?;
Automatic detection of freezing of gait events in patients with Parkinson's disease	Tripoliti, E. E.; Tzallas, A. T.; Tsipouras, M. G.; Rigas, G.; Bougia, P.; Leontiou, M.; Konitsiotis, S.; Chondrogiorgi, M.; Tsouli, S.; Fotiadis, D. I.	2013	Computer Methods and Programs in Biomedicine	110	1	Dec-26		http://dx.doi.org/10.1016/j.cmpb.2012.10.016	Tripoliti 2013	Exclusion reason: Structured assessments (as opposed to free-living); Catherine Morgan (2018-06-21 00:17:16)(Screen): lab-based?;
Accurate Telemonitoring of Parkinsons Disease Progression by Noninvasive Speech Tests	Tsanas, A.; Little, M. A.; McSharry, P. E.; Ramig, L. O.	2010	Ieee Transactions on Biomedical Engineering	57	4	884-893	WOS:000275998200013	10.1109/tbme.2009.2036000	Tsanas 2010	Exclusion reason: Structured assessments (as opposed to free-living); Catherine Morgan (2018-06-21 00:18:21)(Screen): computerised speech lab? (see exclusions);
A data mining methodology for predicting early stage Parkinson's disease using non-invasive, high-dimensional gait sensor data	Tucker, C.; Han, Y.; Black Nembhard, H.; Lee, W. C.; Lewis, M.; Sterling, N.; Huang, X.	2015	IIE Transactions on Healthcare Systems Engineering	5	4	238-254		http://dx.doi.org/10.1080/19488300.2015.1095256	Tucker 2015	Exclusion reason: Wrong setting;
Machine learning classification of medication adherence in patients with movement disorders using non-wearable sensors	Tucker, C. S.; Behoora, I.; Nembhard, H. B.; Lewis, M.; Sterling, N. W.; Huang, X.	2015	Computers in Biology and Medicine	66		120-134		http://dx.doi.org/10.1016/j.compbiomed.2015.08.012	Tucker 2015	Exclusion reason: Structured assessments (as opposed to free-living);

Impact of movement disorders during sleep on patients with Parkinson's disease	Uchino, K.; Shiraishi, M.; Tanaka, K.; Akamatsu, M.; Hasegawa, Y.	2017	Journal of the Neurological Sciences	381	Suppl 1	1044		http://dx.doi.org/10.1016/j.jns.2017.08.2948	Uchino 2017	Exclusion reason: No full text available (abstract/poster only);
Twelve-week sensor assessment in Parkinson's disease: impact on quality of life	Uem, J. M.; Maier, K. S.; Santos, A. T.; Fagerbakke, O.; Larsen, F.; Ferreira, J. J.; Maetzler, W.	2016	Movement disorders Conference: 20th International Congress of Parkinson's Disease and Movement Disorders.		S173			10.1002/mds.26688	Uem 2016	Exclusion reason: No full text available (abstract/poster only);
Twelve-week sensor assessment in Parkinson's disease: Impact on quality of life	van Uem, J. M. T.; Maier, K. S.; Hucker, S.; Scheck, O.; Hobert, M. A.; Santos, A. T.; Fagerbakke, O.; Larsen, F.; Ferreira, J. J.; Maetzler, W.	2016	Movement Disorders	31	9	1337-1338	WOS:000383773600051	10.1002/mds.26676	vanUem 2016	Exclusion reason: Wrong outcomes: e.g., EEG, imaging only, molecular diagnostic/therapeutic techniques, EMG;
Detection of freezing of gait for Parkinson's disease patients based on deep convolutional neural networks	Wang, J.; Liu, Q.; Chen, H.	2017	Chinese Journal of Biomedical Engineering	36	4	418-425		http://dx.doi.org/10.3969/j.issn.0258-8021.2017.04.005	Wang 2017	Exclusion reason: No full text available (abstract/poster only); Catherine Morgan (2019-08-13 06:57:58)(Excluded): Couldn't find a full text in English;
Home Monitoring Musculo-Skeletal Disorders with a Single 3D Sensor	Wang, R. Z.; Medioni, G.; Winstein, C. J.; Blanco, C.; Ieee	2013	2013 Ieee Conference on Computer Vision and Pattern Recognition Workshops			521-528	WOS:000331116100081	10.1109/cvprw.2013.83	Wang 2013	Exclusion reason: Structured assessments (as opposed to free-living);
A Mobile Cloud-Based Parkinson's Disease Assessment System for Home-Based Monitoring	Pan, D.; Dhall, R.; Lieberman, A.; Petitti, D. B.	2015	Jmir Mhealth and Uhealth	3	1		WOS:000359791000029	10.2196/mhealth.3956	Pan 2015	Exclusion reason: Structured assessments (as opposed to free-living);
Diagnosis and monitoring of Parkinson's disease patients based on motor signals	Pastor-Sanz, L.; Arredondo Waldmeyer, M. T.; Cancela Gonzalez, J.; Pastorino, M.; Del Pozo, F.	2012	Parkinsonism and Related Disorders	18	Suppl 2	S102		http://dx.doi.org/10.1016/S1353-8020%2811%29700478-3	Pastor-Sanz 2012	Exclusion reason: No full text available (abstract/poster only); Catherine Morgan (2018-06-20 20:41:21)(Screen): Environment?;
Assessment of Bradykinesia in Parkinson's disease patients through a multi-parametric system	Pastorino, M.; Cancela, J.; Arredondo, M. T.; Pansera, M.; Pastor-Sanz, L.; Villagra, F.; Pastor, M. A.; Martin, J. A.	2011	Conference proceedings: Annual International Conference of the IEEE Engineering in	2011		1810-1813			Pastorino 2011	Exclusion reason: Wrong setting; Catherine Morgan (2018-06-20 20:42:12)(Screen): environment?;

			Medicine and Biology Society							
Home monitoring of patients with Parkinson's disease via wearable technology and a web-based application	Patel, S.; Chen, B. R.; Buckley, T.; Rednic, R.; McClure, D.; Tarsy, D.; Shih, L.; Dy, J.; Welsh, M.; Bonato, P.	2010	Conference proceedings: Annual International Conference of the IEEE Engineering in Medicine and Biology Society	2010		4411-4414			Patel 2010	Exclusion reason: Structured assessments (as opposed to free-living);
Longitudinal monitoring of patients with Parkinson's disease via wearable sensor technology in the home setting	Patel, S.; Chen, B. R.; Mancinelli, C.; Paganoni, S.; Shih, L.; Welsh, M.; Dy, J.; Bonato, P.	2011	Conference proceedings: Annual International Conference of the IEEE Engineering in Medicine and Biology Society	2011		1552-1555			Patel 2011	Exclusion reason: Structured assessments (as opposed to free-living);
Using wearable sensors to predict the severity of symptoms and motor complications in late stage Parkinson's Disease	Patel, S.; Hughes, R.; Huggins, N.; Standaert, D.; Growdon, J.; Dy, J.; Bonato, P.	2008	Conference proceedings: Annual International Conference of the IEEE Engineering in Medicine and Biology Society	2008		3686-3689			Patel 2008	Exclusion reason: Wrong setting; Catherine Morgan (2018-06-20 20:44:28)(Screen): environment?;
SPARK: Personalized Parkinson Disease Interventions through Synergy between a Smartphone and a Smartwatch	Sharma, V.; Mankodiya, K.; De La Torre, F.; Zhang, A.; Ryan, N.; Ton, T. G. N.; Gandhi, R.; Jain, S.	2014	Design, User Experience, and Usability: User Experience Design for Everyday Life Applications and Services, Pt Iii	8519		103-114	WOS:000342848400011		Sharma 2014	Exclusion reason: Structured assessments (as opposed to free-living);
Improved detection of gait abnormalities in Parkinson's disease using an IMU sensor-based system	Shiwani, B.; Roy, S.; Kline, J.; Saint-Hilaire, M.; Thomas, C.; Gennert, M.; De Luca, G.	2017	Movement Disorders	32	Suppl 2	450-451		http://dx.doi.org/10.1002/mds.27087	Shiwani 2017	Exclusion reason: No full text available (abstract/poster only);
Assessment of motor control fluctuations in pd patients under dopaminergic treatment with neuroskill device: Office and home monitoring	Shrairman, R.; Kumar, R.; Godbold, J.; Landau, A.; O'Brien, C.	2015	Neurodegenerative Diseases	15	Suppl 1	185		http://dx.doi.org/10.1159/000381736	Shrairman 2015	Exclusion reason: No full text available (abstract/poster only);

Assessment of fine motor control in Parkinson's disease with the neuroskilltm analysis of handwriting dynamics	Shrairman, R.; O'Brien, C. F.; Landau, A.	2012	Parkinsonism and Related Disorders	18	Suppl 2	S103-S104		http://dx.doi.org/10.1016/S1353-8020%2811%2970482-5	Shrairman 2012	Exclusion reason: Structured assessments (as opposed to free-living);
Longitudinal assessment of fine motor control: Transition from normal control to onset of Parkinson's disease	Shrairman, R.; O'Brien, C.; Landau, A.	2009	Parkinsonism and Related Disorders	15	Suppl 2	S70-S71		http://dx.doi.org/10.1016/S1353-8020%2809%2970285-8	Shrairman 2009	Exclusion reason: No full text available (abstract/poster only);
Freezing of gait and fall detection in Parkinson's disease using wearable sensors: a systematic review	Silva de Lima, A. L. S.; Evers, L. J. W.; Hahn, T.; Bataille, L.; Hamilton, J. L.; Little, M. A.; Okuma, Y.; Bloem, B. R.; Faber, M. J.	2017	Journal of Neurology	264	8	1642-1654	WOS:000406688800009	10.1007/s00415-017-8424-0	deLima 2017	Exclusion reason: Wrong study design;
A Vision-Based System for Movement Analysis in Medical Applications: The Example of Parkinson Disease	Spasojevic, S.; Santos-Victor, J.; Ilic, T.; Milanovic, S.; Potkonjak, V.; Rodic, A.	2015	Computer Vision Systems	9163		424-434	WOS:000364183300038	10.1007/978-3-319-20904-3_38	Spasojevic 2015	Exclusion reason: Structured assessments (as opposed to free-living);
Quantitative assessment of the arm/hand movements in Parkinson's disease using a wireless armband device	Spasojevic, S.; Ilic, T. V.; Stojkovic, I.; Potkonjak, V.; Rodic, A.; Santos-Victor, J.	2017	Frontiers in Neurology	8	AUG	388		http://dx.doi.org/10.3389/fneur.2017.00388	Spasojevic 2017	Exclusion reason: Wrong setting;
What strategies do Parkinson's disease patients use for getting out of bed in the early morning? Preliminary results from home video analysis of 22 patients	Sringean, J.; Anan, C.; Bhidayasiri, R.	2017	Movement Disorders	32	Suppl 2	741-742		http://dx.doi.org/10.1002/mds.27087	Sringean 2017	Exclusion reason: No full text available (abstract/poster only);
How good do Parkinson's disease (PD) patients turn in bed? A comparative study between PD patients and their spouses using multisite accelerometers	Sringean, J.; Taechalertpaisarn, P.; Thanawattano, C.; Bhidayasiri, R.	2014	Movement Disorders	29	Suppl 2	S52-S53			Sringean 2014	Exclusion reason: No full text available (abstract/poster only);
Insights in nocturnal hypokinesia in Parkinson's disease: Quantitative data analysis derived from multisite wearable sensors	Sringean, J.; Taechalertpaisarn, P.; Thanawattano, C.; Bhidayasiri, R.	2015	Movement Disorders	30	Suppl 1	S126-S127		http://dx.doi.org/10.1002/mds.26295	Sringean 2015	Exclusion reason: No full text available (abstract/poster only);
Body position during sleep in patients with	Sringean, J.; Taechalertpaisarn, P.;	2016	Movement Disorders	31	Suppl 2	S676		http://dx.doi.org/10.1002/mds.26688	Sringean 2016	Exclusion reason: No full text available (abstract/poster only);

Parkinson's disease (PD): A sensor-based analysis	Thanawattano, C.; Bhidayasiri, R.									
When is the worst period of nocturnal hypokinesia in Parkinson's disease? A sensor-based analysis	Sringean, J.; Taechalertpaisarn, P.; Thanawattano, C.; Bhidayasiri, R.	2016	Movement Disorders	31	Suppl 2	S680-S681		http://dx.doi.org/10.1002/mds.26688	Sringean 2016	Exclusion reason: No full text available (abstract/poster only);
Identifying balance impairments in people with Parkinson's disease using video and wearable sensors	Stack, E.; Agarwal, V.; King, R.; Burnett, M.; Tahavori, F.; Janko, B.; Harwin, W.; Ashburn, A.; Kunkel, D.	2018	Gait and Posture	62		321-326		http://dx.doi.org/10.1016/j.gaitpost.2018.03.047	Stack 2018	Exclusion reason: Structured assessments (as opposed to free-living);
The cloudUPDRS app: A medical device for the clinical assessment of Parkinson's Disease	Stamate, C.; Magoulas, G. D.; Kueppers, S.; Nomikou, E.; Daskalopoulos, I.; Jha, A.; Pons, J. S.; Rothwell, J.; Luchini, M. U.; Moussouri, T.; Iannone, M.; Roussos, G.	2018	Pervasive and Mobile Computing	43		146-166	WOS:000425086400011	10.1016/j.pmcj.2017.12.005	Stamate 2018	Exclusion reason: Structured assessments (as opposed to free-living);
Deep Learning Parkinson's from Smartphone Data	Stamate, C.; Magoulas, G. D.; Kueppers, S.; Nomikou, E.; Daskalopoulos, I.; Luchini, M. U.; Moussouri, T.; Roussos, G.; Ieee	2017	2017 Ieee International Conference on Pervasive Computing and Communications				WOS:000403607900004		Stamate 2017	Exclusion reason: Structured assessments (as opposed to free-living);
Twenty-four hour non-invasive ambulatory blood pressure and heart rate monitoring in Parkinson's Disease	Stuebner, E.; Vichayanrat, E.; Low, D. A.; Mathias, C. J.; Isenmann, S.; Haensch, C. A.	2013	Frontiers in Neurology	4			WOS:000209629000048	10.3389/fneur.2013.00049	Stuebner 2013	Exclusion reason: Wrong study design;
The Past, Present, and Future of Telemedicine for Parkinson's Disease	Achey, M.; Aldred, J. L.; Aljehani, N.; Bloem, B. R.; Biglan, K. M.; Chan, P.; Cubo, E.; Dorsey, E. R.; Goetz, C. G.; Guttman, M.; Hassan, A.; Khandhar, S. M.; Mari, Z.; Spindler, M.; Tanner, C. M.; van den Haak, P.; Walker, R.; Wilkinson, J. R.; Int Parkinson Movement, Disorder	2014	Movement Disorders	29	7	871-883	WOS:000337670600011	10.1002/mds.25903	Achey 2014	Exclusion reason: Wrong study design;
Vertical ground reaction force marker for Parkinson's disease	Alam, M. N.; Garg, A.; Munia, T. T. K.; Fazel-Rezai, R.; Tavakolian, K.	2017	PLoS One	12	5	e0175951		http://dx.doi.org/10.1371/journal.pone.0175951	Alam 2017	Exclusion reason: Wrong setting; Catherine Morgan (2018-07-02 21:01:26)(Select): Whole-text review: lab environment; Catherine Morgan (2018-06-04 21:00:51)(Screen): Not in home-like environment?;

Clinical feasibility of a wearable, conformable, sensor patch to monitor motor symptoms in Parkinson's disease	Boroojerdi, B.; Claes, K.; Ghaffari, R.; Mahadevan, N.; Markowitz, M.; Melton, K.; Morey, B.; Otoul, C.; Patel, S.; Phillips, J.; Sen-Gupta, E.; Stumpp, O.; Tatla, D.; Wright, J.; Sheth, N.	2017	Movement Disorders	32	Suppl 2	441-443		http://dx.doi.org/10.1002/mds.27087	Boroojerdi 2017	Exclusion reason: No full text available (abstract/poster only); Catherine Morgan (2018-07-04 23:03:30)(Select): Abstract only in MDS, no full text via UoB or google scholar;
Detection of Gait and Postures Using a Miniaturized Triaxial Accelerometer-Based System: Accuracy in Patients With Mild to Moderate Parkinson's Disease	Dijkstra, B.; Kamsma, Y. P.; Zijlstra, W.	2010	Archives of Physical Medicine and Rehabilitation	91	8	1272-1277	WOS:000281178900017	10.1016/j.apmr.2010.05.004	Dijkstra 2010	Exclusion reason: Structured assessments (as opposed to free-living);
Detection, segmentation, and assessment of daily living activities using inertial sensor in people with Parkinson's disease during a cleaning task	Duval, C.; Nguyen, H. P.; Lebel, K.; Bogard, S.; Goubault, E.; Boissy, P.	2018	Parkinsonism and Related Disorders	46	Suppl 2	e83-e84			Duval 2018	Exclusion reason: No full text available (abstract/poster only);
Recent machine learning advancements in sensor-based mobility analysis: Deep learning for Parkinson's disease assessment	Eskofier, B. M.; Lee, S. I.; Daneault, J. F.; Golabchi, F. N.; Ferreira-Carvalho, G.; Vergara-Diaz, G.; Sapienza, S.; Costante, G.; Klucken, J.; Kautz, T.; Bonato, P.	2016	Conference proceedings: Annual International Conference of the IEEE Engineering in Medicine and Biology Society	2016		655-658		http://dx.doi.org/10.1109/EMBC.2016.7590787	Eskofier 2016	Exclusion reason: Wrong setting; Catherine Morgan (2018-06-13 18:02:27)(Screen): unclear whether these assessments took place in home, doesn't sound like it but worth checking ;
Mobility in people with Parkinson's disease with different medication profile	Faucher, G.; Briere, S.; Blamoutier, M.; Roofigari, N.; Nguyen, H. P.; Bogard, S.; Goubault, E.; Duval, C.	2018	Parkinsonism and Related Disorders	46	Suppl 2	e83			Faucher 2018	Exclusion reason: No full text available (abstract/poster only);
Fox insight wear ecosystem: Using mobile technology and cloudbased computing to support PD research	Feldman, I.; Afek, M.; Bataille, L.	2016	Movement Disorders	31	Suppl 2	S675		http://dx.doi.org/10.1002/mds.26688	Feldman 2016	Exclusion reason: No full text available (abstract/poster only);
Stability study of a wearable audio-feedback system for gait rehabilitation in persons with parkinson's disease	Ferrari, A.; Ginis, P.; Mirelman, A.; Gazit, E.; Nieuwboer, A.; Dorfman, M.; Hausdorff, J. M.; Rocchi, L.; Chiari, L.	2015	Gait and Posture	42	Suppl 3	S73		http://dx.doi.org/10.1016/j.gaitpost.2015.03.125	Ferrari 2015	Exclusion reason: No full text available (abstract/poster only);
Ergonomic and human interface design factors for home-based medical	Filipkowski, D. E.; Mera, T. O.; Heldman, D. A.; Giuffrida, J. P.	2011	Movement Disorders	26	Suppl 2	S376		http://dx.doi.org/10.1002/mds.23764	Filipkowski 2011	Exclusion reason: No full text available (abstract/poster only); Catherine Morgan (2018-06-13 18:08:40)(Screen): User feedback

devices in movement disorders										seems to be the main aspect of this - but highly relevant to what we are doing;
Automatic Classification of Tremor Severity in Parkinson's Disease Using a Wearable Device	Jeon, Hyoseon; Lee, Woongwoo; Park, Hyeyoung; Lee, Hong Ji; Kim, Sang Kyong; Kim, Han Byul; Jeon, Beomseok; Park, Kwang Suk	2017	Sensors (Basel, Switzerland)	17	9			https://dx.doi.org/10.3390/s17092067	Jeon 2017	Exclusion reason: Wrong setting; Catherine Morgan (2018-06-13 22:17:49)(Screen): ?home environment;
PAM: Passive marker-based analyzer to test patients with neural diseases	Jobbagy, A.; Hamar, G.; ieee	2004	Proceedings of the 26th Annual International Conference of the Ieee Engineering in Medicine and Biology Society, Vols 1-7	26		4751-4754	WOS:000225461801241		Jobbagy 2004	Exclusion reason: Wrong patient population;
Visualization of spiral drawing data of patients with Parkinson's disease	Jusufi, I.; Nyholm, D.; Memedi, M.	2014	2014 18th International Conference on Information Visualisation			346-350	WOS:000365275200058	10.1109/iv.2014.31	Jusufi 2014	Exclusion reason: Structured assessments (as opposed to free-living); Catherine Morgan (2018-06-13 22:46:14)(Screen): Not requiring direct clinician interaction but structured assessment ?include;
Patient compliance with Parkinson's disease home monitoring system	Filipkowski, D.; Heldman, D.; Espay, A.; Mishra, J.; Mera, T.; Giuffrida, J.	2012	Movement Disorders	27		S94-S95	WOS:000305507700297		Filipkowski 2012	Exclusion reason: No full text available (abstract/poster only);
Objective assessment of motor symptoms in Parkinson's disease using body-worn sensors	Fisher, J. M.; Hammerla, N. Y.; Andras, P.; Rochester, L.; Walker, R. W.	2014	Movement Disorders	29	Suppl 1	S238		http://dx.doi.org/10.1002/mds.25914	Fisher 2014	Exclusion reason: No full text available (abstract/poster only);
Evaluating the acceptability of body-worn sensors to patients with Parkinson's disease	Fisher, J. M.; Hammerla, N. Y.; Rochester, L.; Andras, P.; Walker, R. W.	2014	Movement Disorders	29	Suppl 1	S238		http://dx.doi.org/10.1002/mds.25914	Fisher 2014	Exclusion reason: No full text available (abstract/poster only); Catherine Morgan (2018-07-11 19:39:01)(Select): paper found not exactly same as that quoted - this is an abstract, the full paper was published in TMJ;
Towards the automated detection of near falls during community ambulation in patients with Parkinson's disease	Freedman, T.; Gazit, E.; Brozgol, M.; Giladi, N.; Mirelman, A.; Hausdorff, J. M.	2013	Movement Disorders	28	Suppl 1	S174		http://dx.doi.org/10.1002/mds.25605	Freedman 2013	Exclusion reason: No full text available (abstract/poster only); Catherine Morgan (2018-07-11 19:43:39)(Select): check re full text in uob library;
Wearable motion quantification and electronic diaries for	Hadley, A.; Mendoza, E.; Mennucci, N.; Zimmerman, C.;	2017	Movement Disorders	32	Suppl 2	439-440		http://dx.doi.org/10.1002/mds.27087	Hadley 2017	Exclusion reason: No full text available (abstract/poster only);

long-term monitoring of Parkinson's disease	Giuffrida, J.; Mari, Z.; Burack, M.; Itin, I.; Revilla, F.; Heldman, D.									
Quantative assessment of gait using portable gait rhymogram on music therapy for Parkinson's disease	Hayashi, A.; Mitoma, H.	2012	Parkinsonism and Related Disorders	18	Suppl 2	S16		http://dx.doi.org/10.1016/S1353-8020%2811%2970139-0	Hayashi 2012	Exclusion reason: No full text available (abstract/poster only);
Intra- and multi-day home-based monitoring of Parkinson's disease motor symptoms	Heldman, D. A.; Filipkowski, D.; Espay, A.; Mishra, J.; Giuffrida, J. P.	2012	Movement Disorders	27		S121-S121	WOS:000305507701068		Heldman 2012	Exclusion reason: No full text available (abstract/poster only); Catherine Morgan (2018-06-13 20:39:56)(Screen): Structured assessments (not free-living) but carried out in the home environment ?include;
Wearable sensors and decision algorithms for advanced therapy referral in Parkinson's disease	Heldman, D. A.; Giuffrida, J. P.; Cubo, E.	2016	Movement Disorders	31	Suppl 2	S31		http://dx.doi.org/10.1002/mds.26688	Heldman 2016	Exclusion reason: No full text available (abstract/poster only);
Automated telehealth diagnostics for remote Parkinson monitoring	Heldman, D. A.; Harris, D. A.; Felong, T.; Goldberg, B.; Giuffrida, J. P.; Dorsey, E. R.; Burack, M. A.	2016	Movement Disorders	31	Suppl 2	S186-S187		http://dx.doi.org/10.1002/mds.26688	Heldman 2016	Exclusion reason: Structured assessments (as opposed to free-living); Catherine Morgan (2018-06-13 20:41:06)(Screen): Structured assessments ?include;
Reliability and responsiveness of in-clinic and at-home app-based bradykinesia assessment	Heldman, D. A.; Urrea Mendoza, E.; Lovera, L. C.; Schmerler, D. A.; Giuffrida, J. P.; Espay, A. J.; Garcia, J. X.; Mohammad, M. E.; McFarlane, M. C.; Fernandez, H. H.	2016	Movement disorders Conference: 20th International Congress of Parkinson's Disease and Movement Disorders.			S186		10.1002/mds.26688	Heldman 2016	Exclusion reason: Structured assessments (as opposed to free-living); Catherine Morgan (2018-06-13 20:50:00)(Screen): Duplicated?;
Intra- and Multi-Day Home-Based Monitoring of Parkinson's Disease Motor Symptoms	Heldman, D.; Filipkowski, D.; Espay, A.; Mishra, J.; Giuffrida, J.	2012	Neurology	78			WOS:000303204803433		Heldman 2012	Exclusion reason: No full text available (abstract/poster only); Catherine Morgan (2018-06-13 20:51:26)(Screen): Structured assessments; Catherine Morgan (2018-06-13 20:51:26)(Screen): Structured assessments;
Telehealth management of parkinson's disease using wearable sensors: An exploratory study	Heldman, D.; Harris, D.; Felong, T.; Andrzejewski, K.; Dorsey, E. R.; Giuffrida, J.; Goldberg, B.; Burack, M.	2017	Neurology	88	16 Suppl 1				Heldman 2017	Exclusion reason: Structured assessments (as opposed to free-living); Catherine Morgan (2018-06-13 20:54:06)(Screen): Not the outcomes we are looking for (focussing on the response to the PD outcomes, not their measurements);

Reliability of continuous Parkinson's assessment using wearables	Heldman, D.; Urrea Mendoza, E.; Mennucci, N.; Zimmerman, C.; Giuffrida, J.; Hadley, A.; Mari, Z.; Burack, M.; Itin, I.; Revilla, F.	2017	Movement Disorders	32	Suppl 2	431-432		http://dx.doi.org/10.1002/mds.27087	Heldman 2017	Exclusion reason: No full text available (abstract/poster only);
Automated motion sensor quantification of gait and lower extremity bradykinesia	Heldman, D. A.; Filipkowski, D. E.; Riley, D. E.; Whitney, C. M.; Walter, B. L.; Gunzler, S. A.; Giuffrida, J. P.; Mera, T. O.	2012	Conference proceedings: Annual International Conference of the IEEE Engineering in Medicine and Biology Society	2012		1956-1959			Heldman 2012	Exclusion reason: Structured assessments (as opposed to free-living); Catherine Morgan (2018-06-13 20:33:41)(Screen): Unclear whether the home-based system is used in this study?;
Wearable sensors for advanced therapy referral in Parkinson's disease	Heldman, D. A.; Giuffrida, J. P.; Cubo, E.	2016	Journal of Parkinson's Disease	6	3	631-638		http://dx.doi.org/10.3233/JPD-160830	Heldman 2016	Exclusion reason: Structured assessments (as opposed to free-living);
Telehealth Management of Parkinson's Disease Using Wearable Sensors: An Exploratory Study	Heldman, Dustin A.; Harris, Denzil A.; Felong, Timothy; Andrzejewski, Kelly L.; Dorsey, E. R; Giuffrida, Joseph P.; Goldberg, Barry; Burack, Michelle A.	2017	Digital biomarkers	1	1	43-51		https://dx.doi.org/10.1159/000475801	Heldman 2017	Exclusion reason: Structured assessments (as opposed to free-living); Catherine Morgan (2018-06-13 20:56:12)(Screen): duplicate? also, not measuring PD outcomes, looking at clinical responses to them;
Automated Parkinson's disease motor assessment for clinical and ambulatory monitoring	Heldman, P.; Espay, A.; Lewitt, P.; Giuffrida, J.	2009	Parkinsonism and Related Disorders	15	Suppl 2	S63		http://dx.doi.org/10.1016/S1353-8020%2809%2970257-3	Heldman 2009	Exclusion reason: Structured assessments (as opposed to free-living); Catherine Morgan (2018-06-13 20:57:20)(Screen): Structured assessments at home, not free-living;
Evaluation of the overall system precision of the Welch-Allyn transtelephonic home blood pressure monitor in adults with Parkinson's disease	Herbst, T.; Anis-Anwar, Y.; Giacco, S.; White, W. B.	2002	Blood Pressure Monitoring	7	5	285-288	WOS:000179179300007	10.1097/00126097-200210000-00007	Herbst 2002	Exclusion reason: Wrong setting;
Application of an instrumented 3-day activity monitor to evaluate Parkinson's disease clinical subtypes	Herman, T.; Weiss, A.; Marina, B.; Giladi, N.; Hausdorff, J. M.	2013	Movement Disorders	28	Suppl 1	S322		http://dx.doi.org/10.1002/mds.25605	Herman 2013	Exclusion reason: No full text available (abstract/poster only);
Gait and balance in Parkinson's disease subtypes: objective measures and classification considerations	Herman, T.; Weiss, A.; Brozgol, M.; Giladi, N.; Hausdorff, J. M.	2014	Journal of Neurology	261	12	2401-2410	WOS:000345383800019	10.1007/s00415-014-7513-6	Herman 2014	Exclusion reason: Structured assessments (as opposed to free-living);

Walk on the wild side: the complexity of free-living mobility assessment	Hickey, Aodhan; Stuart, Sam; O'Donovan, Karol; Godfrey, Alan	2017	Journal of epidemiology and community health	71	6	624		https://dx.doi.org/10.1136/jech-2016-208752	Hickey 2017	Exclusion reason: Wrong study design; Catherine Morgan (2018-06-13 20:47:14)(Irrelevant): Probably a commentary or review, but worth a look?;
Evaluating Parkinson's disease patients at home: Utility off self-videotaping for objective motor, dyskinesia and ON/OFF assessments	Hinson, V.; Goetz, C. K.; Leurgans, S.; Blasucci, L. M.; Zimmerman, J. L.; Fan, W. Q.; Nguyen, T.; Hsu, A.	2008	Neurology	70	11	A56-A57	WOS:000257197200242		Hinson 2008	Exclusion reason: Structured assessments (as opposed to free-living);
Long-term monitoring gait analysis using a wearable device in daily lives of patients with Parkinson's disease: The efficacy of selegiline hydrochloride for gait disturbance	Iijima, M.; Mitoma, H.; Uchiyama, S.; Kitagawa, K.	2017	Frontiers in Neurology	8	OCT	542		http://dx.doi.org/10.3389/fneur.2017.00542	Iijima 2017	Exclusion reason: Wrong setting; Catherine Morgan (2018-06-13 21:18:37)(Screen): unclear if assessments done at home or in lab;
SENSE-PARK: Measuring Parkinson's disease in the home environment in an objective, continuous and minimally obtrusive fashion	Isaacs, T.; Matthews, H.; Duffen, J.; Al-Jawad, A.; Larsen, F.; Serrano, A.; Weber, P.; Thoms, A.; Sollinger, S.; Ferreira, J. J.; Domingos, J.; Santos, A. T.; Heger, T.; Maetzler, W.; Graessner, H.	2013	Journal of Parkinson's Disease	3	Suppl 1	169-170		http://dx.doi.org/10.3233/JPD-139905	Isaacs 2013	Exclusion reason: No full text available (abstract/poster only);
Activity Recognition Using Multiple Inertial Measurement Units	Jalloul, N.; Poree, F.; Viardot, G.; L'Hostis, P.; Carrault, G.	2016	IRBM	37	3	180-186		http://dx.doi.org/10.1016/j.irbm.2016.02.008	Jalloul 2016	Exclusion reason: Structured assessments (as opposed to free-living); Catherine Morgan (2018-06-13 21:27:12)(Screen): ?home environment;
Automatic assestment of levodopa-induced dyskinesias in daily life by neural networks	Keijsers, N. L. W.; Horstink, M. W. I. M.; Gielen, S. C. A. M.	2003	Movement Disorders	18	1	70-80		http://dx.doi.org/10.1002/mds.10310	Keijsers 2003	Exclusion reason: Structured assessments (as opposed to free-living);
Automatic assessment of levodopa-induced dyskinesias in daily life by neural networks	Keijsers, N. L. W.; Horstink, Mwim; Gielen, Scam	2003	Movement Disorders	18	1	70-80	WOS:000180402600008	10.1002/mds.10310	Keijsers 2003	Exclusion reason: Structured assessments (as opposed to free-living);
Movement parameters that distinguish between voluntary movements and levodopa-induced dyskinesia in Parkinson's disease	Keijsers, N. L. W.; Horstink, M. W. I. M.; Gielen, S. C. M.	2003	Human Movement Science	22	1	67-89		http://dx.doi.org/10.1016/S0167-9457%2802%290179-3	Keijsers 2003	Exclusion reason: Structured assessments (as opposed to free-living);
Ambulatory motor assessment in Parkinson's disease	Keijsers, N. L. W.; Horstink, M. W. I. M.; Gielen, S. C. A. M.	2006	Movement Disorders	21	1	34-44		http://dx.doi.org/10.1002/mds.20633	Keijsers 2006	Exclusion reason: Structured assessments (as opposed to free-living);

Regularization in finite mixture of regression models with diverging number of parameters	Khalili, A.; Lin, S.	2013	Biometrics	69	2	436-446		http://dx.doi.org/10.1111/biom.12020	Khalili 2013	Exclusion reason: Wrong outcomes: e.g., EEG, imaging only, molecular diagnostic/therapeutic techniques, EMG;
A Wearable Accelerometer System for Unobtrusive Monitoring of Parkinson's Disease Motor Symptoms	Khan, F. M.; Barnathan, M.; Montgomery, M.; Myers, S.; Cote, L.; Loftus, S.; Ieee	2014	2014 IEEE International Conference on Bioinformatics and Bioengineering			120-125	WOS:000380460200018	10.1109/bibe.2014.18	Khan 2014	Exclusion reason: Structured assessments (as opposed to free-living); Catherine Morgan (2018-06-13 22:58:11)(Screen): ?environment;
Assessment of PD speech anomalies @ home	Khan, T.; Westin, J.	2011	Movement Disorders	26	Suppl 2	S359		http://dx.doi.org/10.1002/mds.23764	Khan 2011	Exclusion reason: Established speech recognition systems; Catherine Morgan (2018-06-13 23:00:52)(Screen): Perhaps not include as this is a speech processing system?;
Validation of Freezing-of-Gait Monitoring Using Smartphone	Kim, H. B.; Lee, H. J.; Lee, W. W.; Kim, S. K.; Jeon, H. S.; Park, H. Y.; Shin, C. W.; Yi, W. J.; Jeon, B.; Park, K. S.		Telemedicine and E-Health				WOS:000431103100001	10.1089/tmj.2017.0215		Exclusion reason: No full text available (abstract/poster only); Catherine Morgan (2018-06-14 00:14:28)(Screen): Unclear environment ?home;
Assessing abnormal gaits of parkinson's disease patients using a wearable motion detector	Su, R. H.; Hsu, Y. L.; Chan, L.; Lin, H.; Yang, C. C.	2014	Biomedical Engineering - Applications, Basis and Communications	26	2	1450031		http://dx.doi.org/10.4015/S1016237214500318	Su 2014	Exclusion reason: Structured assessments (as opposed to free-living);
WiiPD--an approach for the objective home assessment of Parkinson's disease	Synnott, J.; Chen, L.; Nugent, C. D.; Moore, G.	2011	Conference proceedings: Annual International Conference of the IEEE Engineering in Medicine and Biology Society	2011		2388-2391			Synnott 2011	Exclusion reason: Structured assessments (as opposed to free-living);
WiiPD-Objective Home Assessment of Parkinson's Disease Using the Nintendo Wii Remote	Synnott, J.; Chen, L. M.; Nugent, C. D.; Moore, G.	2012	Ieee Transactions on Information Technology in Biomedicine	16	6	1304-1312	WOS:000312268300036	10.1109/titb.2012.2215878	Synnott 2012	Exclusion reason: Structured assessments (as opposed to free-living);
Overnight accelerometric monitoring of inability to turn in bed in Parkinson's disease and hemiplegic stroke	Tanaka, K.; Shiraishi, M.; Uchino, K.; Akamatsu, M.; Hasegawa, Y.	2017	Journal of the Neurological Sciences	381	Suppl 1	1035		http://dx.doi.org/10.1016/j.jns.2017.08.2921	Tanaka 2017	Exclusion reason: No full text available (abstract/poster only);
PDSS: A novel mobile-based Parkinson's disease severity score	Zhan, A.; Mohan, S.; Elson, M.; Dorsey, E.; Terzis, A.; Saria, S.	2017	Movement Disorders	32	Suppl 2	437-439		http://dx.doi.org/10.1002/mds.27087	Zhan 2017	Exclusion reason: Structured assessments (as opposed to free-living); Catherine Morgan (2018-

										06-21 01:05:37)(Screen): environment?;
Quantification assessment of bradykinesia in Parkinson's disease based on a wearable device	Zhirong, Lin; Houde, Dai; Yongsheng, Xiong; Xuke, Xia; Shi-Jinn, Horng	2017	Conference proceedings: Annual International Conference of the IEEE Engineering in Medicine and Biology Society	2017		803-806		https://dx.doi.org/10.1109/EMBC.2017.8036946	Zhirong 2017	Exclusion reason: Structured assessments (as opposed to free-living); Catherine Morgan (2018-06-21 01:07:24)(Screen): environment?;
Comparing wearable activity sensors and self-report measures of mobility	Zhu, L. F.; Boissy, P.; Lavigne-Pelletier, C.; Jog, M.; Edwards, R.; Duval, C.; Speechley, M.	2015	Movement Disorders	30	Suppl 1	S474		http://dx.doi.org/10.1002/mds.26295	Zhu 2015	Exclusion reason: No full text available (abstract/poster only);
Improving mobility assessment of Parkinson's patients using the WIMuGPS system	Zhu, L. F.; Boissy, P.; Lavigne-Pelletier, C.; Jog, M.; Edwards, R.; Duval, C.; Speechley, M.	2015	Movement Disorders	30	Suppl 1	S473-S474		http://dx.doi.org/10.1002/mds.26295	Zhu 2015	Exclusion reason: No full text available (abstract/poster only);
Quantitative assessment of home and community mobility of persons with Parkinson disease and their spousal caregivers	Zhu, L.; Lavigne-Pelletier, C.; Blamoutier, M.; Briere, S.; Dibsdale, M.; Boissy, P.; Jog, M.; Duval, C.; Speechley, M.	2013	Journal of Parkinson's Disease	3	Suppl 1	176-177		http://dx.doi.org/10.3233/JPD-139905	Zhu 2013	Exclusion reason: No full text available (abstract/poster only);
A Computer Vision-Based System for Stride Length Estimation using a Mobile Phone Camera	Zhu, W.; Anderson, B.; Zhu, S. G.; Wang, Y.; Acm	2016	Assets'16: Proceedings of the 18th International Acm Sigaccess Conference on Computers and Accessibility			121-130	WOS:000387862800014	10.1145/2982142.2982156	Zhu 2016	Exclusion reason: Structured assessments (as opposed to free-living); Catherine Morgan (2018-06-21 01:09:08)(Screen): environment? 'real clinical' - what is this?;
